# Tryptophan Metabolism in Central Nervous System Diseases: Pathophysiology and Potential Therapeutic Strategies

**DOI:** 10.14336/AD.2022.0916

**Published:** 2023-06-01

**Authors:** Yinrou Huang, Mengke Zhao, Xuemei Chen, Ruoyu Zhang, Anh Le, Michael Hong, Yufei Zhang, Lin Jia, Weidong Zang, Chao Jiang, Junmin Wang, Xiaochong Fan, Jian Wang

**Affiliations:** ^1^Department of Pain Medicine, The First Affiliated Hospital of Zhengzhou University, Zhengzhou, China.; ^2^Department of Human Anatomy, School of Basic Medical Sciences, Zhengzhou University, Zhengzhou, Henan, China.; ^3^Program in the McKelvey School of Engineering, Washington University in St. Louis, Saint Louis, MO 63130, USA.; ^4^Department of Internal Medicine, Sinai Hospital of Baltimore, Baltimore, MD 21215, USA.; ^5^Department of Economics, University of Washington, WA 98125, USA.; ^6^Department of Neurology, The Fifth Affiliated Hospital of Zhengzhou University, Zhengzhou, Henan, China

**Keywords:** central nervous system disease, depression, kynurenine pathway, methoxyindole pathway, neuroinflammation, tryptophan

## Abstract

The metabolism of L-tryptophan (TRP) regulates homeostasis, immunity, and neuronal function. Altered TRP metabolism has been implicated in the pathophysiology of various diseases of the central nervous system. TRP is metabolized through two main pathways, the kynurenine pathway and the methoxyindole pathway. First, TRP is metabolized to kynurenine, then kynurenic acid, quinolinic acid, anthranilic acid, 3-hydroxykynurenine, and finally 3-hydroxyanthranilic acid along the kynurenine pathway. Second, TRP is metabolized to serotonin and melatonin along the methoxyindole pathway. In this review, we summarize the biological properties of key metabolites and their pathogenic functions in 12 disorders of the central nervous system: schizophrenia, bipolar disorder, major depressive disorder, spinal cord injury, traumatic brain injury, ischemic stroke, intracerebral hemorrhage, multiple sclerosis, Alzheimer’s disease, Parkinson’s disease, amyotrophic lateral sclerosis, and Huntington’s disease. Furthermore, we summarize preclinical and clinical studies, mainly since 2015, that investigated the metabolic pathway of TRP, focusing on changes in biomarkers of these neurologic disorders, their pathogenic implications, and potential therapeutic strategies targeting this metabolic pathway. This critical, comprehensive, and up-to-date review helps identify promising directions for future preclinical, clinical, and translational research on neuropsychiatric disorders.

## 1. Introduction

**Figure 1.**
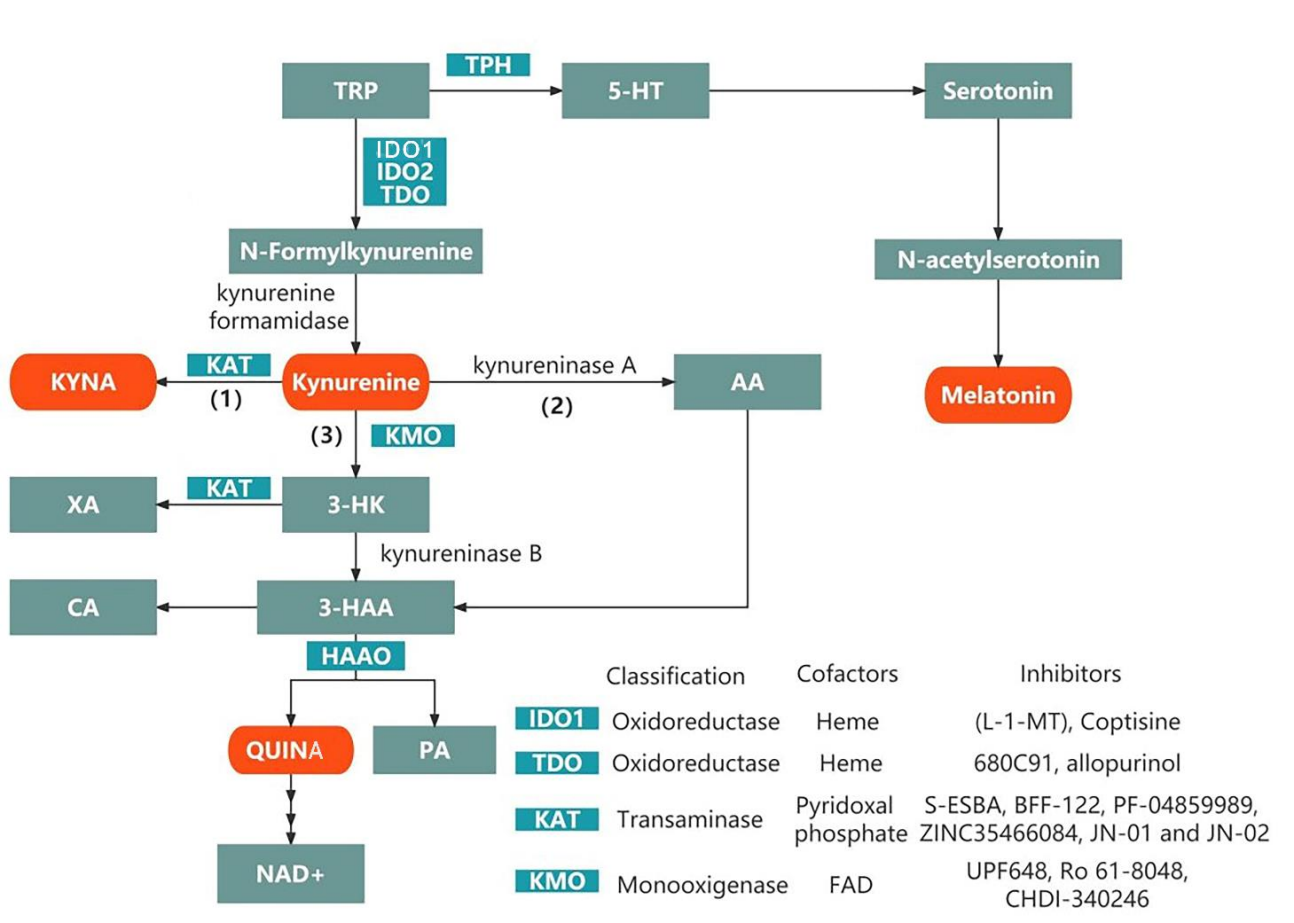
Tryptophan metabolism by the kynurenine and methoxyindole pathways. Kynurenine is a central KP metabolite capable of degradation through three specific pathways, shown in (1), (2) and (3) in the schematic diagram, to generate different neuroactive metabolites. Abbreviations: TRP, tryptophan; IDO, indoleamine-2,3-dioxygenase; TDO, tryptophan-2,3-dioxygenase; KAT, kynurenine aminotransferase I-III; AA, anthranilic acid; 3-HK, 3-hydroxykynurenine; 3-HAA, 3-hydroxyanthrenillc acid; KMO, kynurenine 3-monooxygenase; HAAO, 3-hydroxyanthranilate 3,4-dioxygenase; KP, kynurenine pathway; KYNA, kynurenic acid; PA, picolinic acid; QUINA, quinolinic acid; TPH, tryptophan hydroxylase; CA, cinnabarinic acid; XA, xanthurenic acid; NAD^+^, nicotinamide adenine dinucleotide; 5-HT, 5-hydroxytryptophan; TPH, tryptophan hydroxylase.

L-tryptophan (TRP) is an essential amino acid that must be obtained from the diet. The TRP metabolites of the kynurenine pathway (KP) or the methoxyindole pathway (MP) are involved in the biosynthesis of proteins that are essential for neuronal construction and maintenance. These metabolites are significantly associated with the appearance, development, and outcomes of various neuropsychiatric disorders [1]. The metabolism of TRP by KP and MP is described in Figure 1. We first review changes in TRP metabolism and its pathogenic role in psychiatric disorders, such as schizophrenia (SCZ), bipolar disorder (BD), and major depressive disorder (MDD) [2], and in disorders of the central nervous system (CNS), such as spinal cord injury (SCI) [3], traumatic brain injury (TBI), ischemic stroke, and intracerebral hemorrhage (ICH) [4]. Next we review the role of TRP in multiple sclerosis (MS), an autoimmune disease [5], and neurodegenerative disorders such as Alzheimer’s disease (AD), Parkinson’s disease (PD), amyotrophic lateral sclerosis (ALS), and Huntington’s disease (HD) [6]. We also review preclinical and clinical studies published mainly since 2015 that explore TRP metabolism, its downstream metabolic molecules, relevant biomarkers, and the potential therapeutic targets of this signaling pathway. This review is pivotal, comprehensive, and up to date and provides new ideas for exploring new treatment options and identifying promising directions for future research on drug discovery of neuropsychiatric disorders.

## 2. The KP

**Figure 2.**
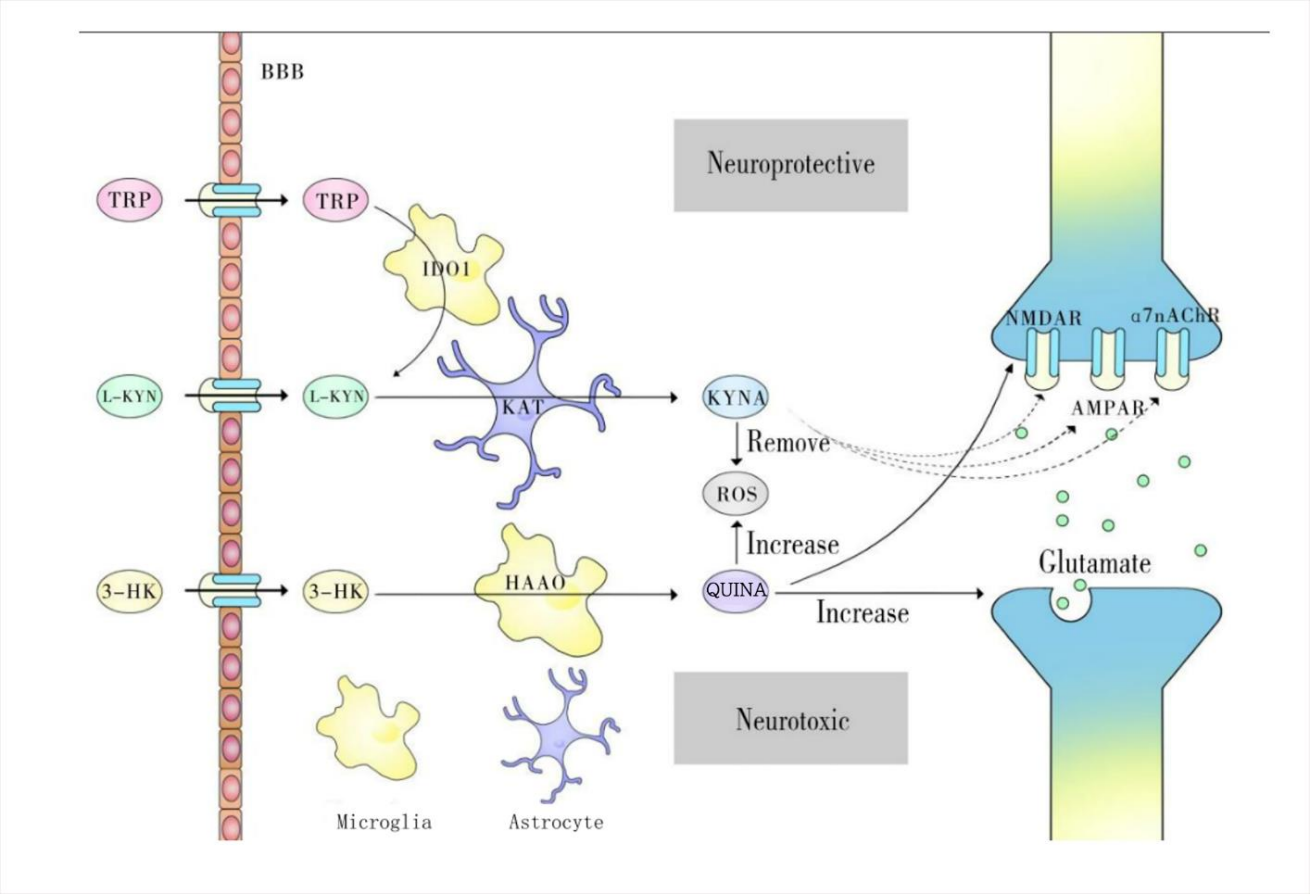
The main branches of the kynurenine pathway. TRP, L-KYN, and 3-HK can penetrate the BBB. Thus, they are converted to different intermediates in the extracellular space of brain tissue. TRP is converted to L-KYN by IDO1 in macrophages. L-KYN is converted to KYNA in astrocytes. 3-HK is converted to QUINA by HAAO in microglia. In diseases other than SCZ, KYNA is neuroprotective, as it can remove ROS and inhibit NMDARs, α7nAch, and AMPARs. QUIN is neurotoxic, as it can increase ROS formation, increase glutamate production, and activate NMDARs. Abbreviations: TRP, tryptophan; 3-HK, 3-hydroxykynurenine; HAAO, 3-hydroxyanthranilate 3,4-dioxygenase; α7nAChR: α7 nicotinic acetylcholine receptor; AMPAR: α-amino-3-hydroxy-5-methyl-4-isoxazole-propionic acid receptor; BBB: blood-brain barrier; IDO, indoleamine-2,3-dioxygenase; KAT, kynurenine aminotransferases I-III; KMO, kynurenine 3-monooxygenase; KYNA, kynurenic acid; L-KYN, L-kynurenine; NMDAR: N-methyl-D-aspartic acid receptor; TRP, tryptophan; QUINA, quinolinic acid; SCZ, schizophrenia; ROS, reactive oxygen species

TRP is absorbed from the intestinal tract and transported to the CNS via neutral amino acid transporters on the blood-brain barrier (BBB) [7]. Under physiological conditions, more than 95% of free TRP is metabolized to kynurenine through the KP. In addition, a small portion is converted to serotonin and other metabolites [8]. The main branches of the KP are described in Figure 2. In KP, L-kynurenine (L-KYN), anthranilic acid (AA), 3-hydroxykynurenine (3-HK), and kynurenic acid (KYNA) are formed in the brain and peripheral tissues. Peripheral kynurenine and other metabolites (excluding KYNA and 3-hydroxyanthranilic acid (3-HAA)) are involved in central KP metabolism by passing through the BBB [9, 10].

In KP, the first step is the conversion of TRP to N-formyl kynurenine by indoleamine 2,3-dioxygenase (IDO1 or IDO2) and tryptophan-2,3-dioxygenase (TDO) and then to L-KYN. IDO1 is expressed, in addition to the brain, in many other organs, such as the kidney, pancreas, and white blood cells [11]. IDO1 plays a critical pathogenic role in brain disorders when activated by proinflammatory cytokines [12], such as interferon γ (IFN-γ), interleukin-6 (IL-6), and tumor necrosis factor α (TNF-α) [13]. This effect is more prominent in women than in men [14]. Dendritic cells also express IDO1 under physiological conditions but are highly induced by traumatic, autoimmune, and neuroinflammatory diseases [15-17]. IDO1 expression has been reported to be upregulated 6 h after injury and remains elevated for up to 21 days in a controlled cortical impact model of pediatric TBI in rabbits [18]. Unlike IDO1, IDO2 is not active in TRP decomposition. Its physiological functions and roles in disease conditions involving KP activity are not yet clear [19]. However, Lauren M F Merlo et al. showed that IDO2 plays a proinflammatory role in mediating B and T-cell activation in immune responses [20]. TDO is present mainly in the liver [21]. The peripheral system produces the majority of L-KYN (approximately 60%), but not all KYN metabolites are permeable to the brain. The rest is generated in the brain [22].

The KP pathway mainly produces two biologically active metabolites, quinolinic acid (QUINA) and KYNA. Kynurenine 3-monooxygenase (KMO) is predominantly expressed in microglia [23]. KMO converts KYN to 3-HK, which is metabolized into the potential neurotoxin QUINA [24]. These two products antagonize each other and reach a balance. Neuroprotective L-KYN has immunosuppressive properties that inhibit antigen-presenting cell (APC) activities, especially in dendritic cells [25]. Furthermore, L-KYN blocks T-cell proliferation, upregulates programmed cell death-1, and leads to the generation of regulatory T cells [26]. Therefore, L-KYN exhibits neuroprotective and anti-inflammatory properties by regulating innate and adaptive immunity. In a model of inflammation-induced depression, KYN increased at 2 h, and KYN concentrations decreased back to baseline levels at 6 h [27]. The concentration of QUINA in the cerebrospinal fluid of adult patients with TBI increases significantly in the first 72-83 h after injury, reaching approximately 9 times the normal concentration of QUINA. KYN levels in cerebrospinal fluid from patients with severe TBI increases on Days 4 and 5 after injury, KYNA increases on Days 2-5 after injury, and QUINA increases on Days 1-5 after injury [24]. Zakhary G et al. found that the expression of QUINA and KYNA increased at 24 and 72 h after partial frontal lobectomy in rats [28].

Three branches are involved in the metabolism of kynurenine: (1) the KYNA pathway, (2) the AA pathway, and (3) the 3-HK pathway. Next, 3-HK is converted to 3-HAA, which merges with the second metabolic pathway. Finally, 3-HAA can be converted to QUINA and eventually nicotinamide adenine dinucleotide (NAD+) or picolinic acid (PA) [19]. The primary metabolites in the TRP pathway are described in Table 1.

**Table 1:** The primary metabolites in the tryptophan pathway.

Substances	Structural formula	Functions in the Kynurenine pathway
TRP	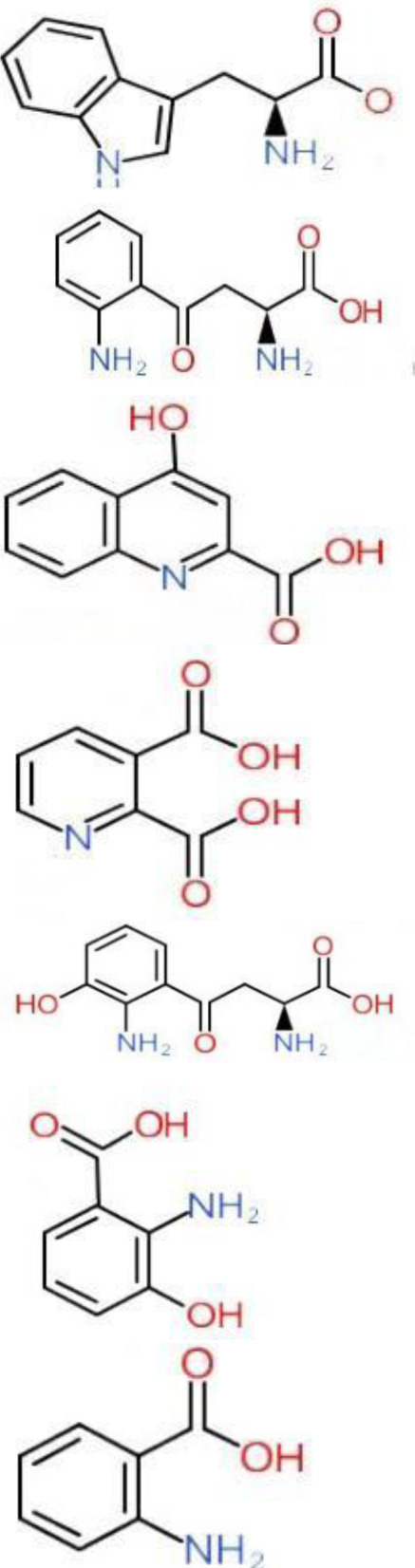	The metabolic substrate of KP pathway
L-KYN		1. Neuroprotective effect 2. Immunosuppression, inhibition of the activity of natural killer cells, APC and DC [25] 3. Blocking T-cell proliferation and upregulation of regulatory T cells [43]
KYNA		1. Neuroprotective effect 2. A competitive antagonist of NMDA and AMPA [30] 3. Anti-inflammatory effect by stimulating GPR35 [34] 4. Immunosuppressive effect by activating AhR [37] 5. An antioxidant to remove ROS [31]
QUINA		1. Neural excitotoxicity [6] 2. Selective activation of NMDA receptors [8] 3. ROS formation and lipid peroxidation [29] 4. BBB disruption [47] 5. Upregulation of nitric oxide synthase and increases in neurotoxicity [93]
3-HK		1. The metabolite of L-KYN catalyzed by KMO 2. Neurotoxic effect 3. Producing free radicals and participating in the metabolism of oxidative stress and fat peroxidation [22]
3-HAA		1. The 3-HK metabolite catalyzed by Kynureninase 2. Neurotoxic effect
AA		1. The metabolite of L-KYN catalyzed by Kynureninase 2. Inhibition of 3-HAA metabolism in QUINA and PA, thus exhibiting a neuroprotective effect [161]

Abbreviations: 3-HAA: 3-hydroxyanthrenillc acid; 3-HK: 3-hydroxykynurenine; AA: anthranilic acid; AhR: aryl hydrocarbon receptor; AMPA: α-amino-3-hydroxy-5-methyl-4-isoxazole-propionic acid; APC: antigen-presenting cells; BBB: blood-brain barrier; DC: dendritic cells; GPR35: G protein-coupled receptor 35; KMO: kynurenine 3-monooxygenase; KP: kynurenine pathway; KYNA: kynurenic acid; L-KYN: L-kynurenine; NMDA: N-methyl-D-aspartic acid; PA: picolinic acid; QUINA: quinolinic acid; ROS: reactive oxygen species; TRP: L-tryptophan.

The three branches ultimately generate two metabolites: KYNA and QUINA [29]. However, acute injuries or chronic neuroinflammation disrupt this equilibrium and cause neurotoxic metabolites to predominate. These toxic metabolites can damage the CNS and cause neurodegeneration. In contrast to previous research, recent studies have shown that KYNA might exert both neuroprotective and neurotoxic effects [19].

(1) L-KYN generates KYNA by kynurenine amino-transferase (KAT). KAT enzymes are believed to be expressed in brain astrocytes. KYNA inhibits endogenous broad-spectrum glutamate receptors, namely, N-methyl-D-aspartic acid receptors (NMDAR), kainite receptors, and α-amino-3-hydroxy-5-methyl-4-isoxazole-propionic acid receptors (AMPAR) [30]. The most significant effect is an inhibition of the NMDAR glycine binding site at high micromolar concentrations [31]. KYNA is also a partial antagonist of the ionic glutamate receptor and the kainite receptor, inhibiting the effect of glutamate at high concentrations [30]. Significantly, nanomolar to micromolar levels of KYNA can facilitate AMPA receptor responses [32]. Furthermore, KYNA can function as a noncompetitive antagonist of the α7 nicotinic acetylcholine receptor (α7nAchR) [33]. It interferes with glutamate and nicotinic neurotransmission, reducing glutamate levels in neuronal synapses and thus preventing excessive glutamate excitement. Excess glutamate in the synaptic cleft dysregulates Ca2+ homeostasis, mitochondrial function, and reactive oxygen species (ROS) production [5]. KYNA also stimulates G protein coupled receptor (GPR) 35, which is expressed on the surface of various immune cells and exhibits an anti-inflammatory effect [34]. For example, the KYNA-GPR35 interaction can reduce the inflammatory response of mononuclear macrophages to lipopolysaccharide [35] and release cytokines from human invariant natural killer cells [36]. Similarly, KYNA activates the aryl hydrocarbon receptor (AhR), which may exert an immunosuppressive effect similar to that of KYN [37]. One recent study demonstrated that KYNA removes ROS as an antioxidant [31]. This data indicate that KYNA protects against neurotoxicity by inhibiting NMDAR. In addition, KYNA antagonism to NMDA and α7nAch exacerbates cognitive dysfunction in SCZ [38].

(2) L-KYN is degraded by kynurenase to produce AA, which is further converted to 3-HAA by monohydroxylase. Serum AA levels may indicate the severity of depression, and brain AA concentrations are significantly associated with the appearance of depression during the treatment of patients with hepatitis C [9].

(3) L-KYN can be catalyzed by KMO to 3-HK and then converted to 3-HAA by KYNU, therefore merging with the second metabolic branch. A series of enzymes, such as 3-hydroxyanthranilate oxidase, metabolize 3-HAA to QUINA and ultimately to NAD^+^ or PA. In addition, 3-HK can be converted to xanthurenic acid (XA) by KAT. Finally, 3-HAA is oxidized to generate cinnabarinic acid (CA).

KMO is expressed in resident macrophages in adipose tissue [39] and predominantly in microglia [40] but not in neurons or astrocytes in the brain [41, 42] In TBI or neurodegenerative conditions, the main rate-limiting enzyme in neurotoxicity, KMO, is activated by proinflammatory factors in microglia to generate neurotoxic products such as 3-HK, 3-HAA, and QUINA [12]. Therefore, KMO is considered a promising target for anti-inflammatory therapy to treat neuroinflammation, but modulation of the dual role of KMO remains to be studied.

3-HK is a product of L-KYN transformed through KMO, which has strong lipophilicity and can easily pass through the BBB [43]. 3-HK autoxidation converts it to 3-HAA and participates in fat peroxidation and other oxidative stress processes [22].

QUINA can mediate neural excitotoxicity by promoting glutamate production and inhibiting the uptake and conversion of glutamate into glutamine [44]. However, QUINA can also selectively activate NMDARs [8]. This effect is more pronounced in the hippocampus, neocortex, and striatum but not in the cerebellum or spinal cord neurons. Neurons in the former locations predominantly express the NR2B subunit of NMDAR, while neurons in the latter brain areas mainly contain the NR2C subunits, and QUINA has a higher affinity for the NR2B subunits [45]. In addition, QUINA can combine with iron to transfer electrons to oxygen to form ROS, which leads to lipid peroxidation [29]. This suggests the use of iron chelation to inhibit the binding of QUINA to iron and thus protect neurons [46]. QUINA also affects GFAP phosphorylation and decreases the stability of the astrocyte cytoskeleton, thus contributing to the breakdown of the BBB [47].

Furthermore, by activating NMDARs, QUINA induces astrocyte apoptosis and neuronal dysfunction [47]. QUINA also increases the expression of nitric oxide synthase and thus influences the production of nitric oxide (NO•) as well as vasodilation [48]. The effects of QUINA and KYNA show a certain degree of antagonism, suggesting that the balance of these two metabolites may affect the development and prognosis of various neurological diseases. Furthermore, QUINA generates NAD^+^ under physiological conditions (< 100 nm). It is also involved in energy metabolism within mitochondria [47]. The imbalance in the NAD^+^/NADH ratio is associated with mitochondrial disorders and aging and age-related diseases [3].

CA and XA act on metabotropic glutamate receptors (mGlu) [30]. CA can inhibit excitotoxic neuronal cell death by activating mGlu4 receptors, while XA has antipsychotic-like effects by positively activating mGlu2 and mGlu3 receptors [49]. In addition, CA can serve as an AhR ligand to stimulate T cells to produce IL-22[50]. Changes caused by the combination of CA and AhR are not yet known. Xanthine may exhibit a neuroprotective effect by reducing 3-OH-kynurenine levels. It also induces cell apoptosis by altering mitochondrial function and increasing the concentration of intracellular calcium ions, thus playing an excitatory role in the brain [51]. Under physiological conditions, PA is rarely present in the brain, replacing NAD^+^ when enzymes such as 3-hydroxyanthranilate oxidases are present [34]. As a secondary signal for macrophages, PA causes the activation of IFN-γ-primed macrophages and triggers a cytokine-driven inflammatory response [52].

## 3. The MP

Another metabolic direction of TRP is through the MP. First, tryptophan-5-hydroxylase metabolizes TRP to 5-hydroxy tryptophan (5-HT). It is then converted to serotonin by the aromatic L-amino acid decarboxylase enzyme. Next, serotonin forms N-acetyl serotonin (NAS), also an agonist of BDNF receptors [53], and melatonin, by an alkylamine N-acetyltransferase.

Melatonin is neuroprotective against glutamate toxicity [54]. Previous studies have shown multiple anti-inflammatory effects, including inhibition of the expression of neuronal NO synthase and cyclooxygenase-2, reducing the BOX-1 signal of the high-mobility group and activating Toll-like receptor-4, negatively regulating NLRP3, inhibiting the activation of NF-κB, and positively regulating nuclear factor erythroid 2-related factor 2 [55]. Melatonin penetrates the BBB [56] and thus binds to G protein-coupled receptors after secretion, namely, MT1 and MT2 [57]. As an antioxidant, it directly scavenges free radicals and indirectly stimulates antioxidant enzymes to inhibit oxidative enzymes and stabilize mitochondrial membranes [58, 59]. Furthermore, the cascade reaction products of melatonin have antioxidant properties. N(1)-acetyl-5-methoxykynuramine, for example, has an efficient antioxidant effect [60]. In the Fenton/Haber-Weiss reaction, melatonin chelates ferrous iron and copper, reducing toxic hydroxyl radicals and oxidative stress [61]. Melatonin is said to be an efficient antioxidant and protects against metal-induced oxidative stress; it also exhibits significant antineuroinflammatory properties and can protect against AD [62]. Melatonin has been investigated in several clinical trials for its antioxidant, anti-inflammatory, and antiapoptotic properties and the restoration of tissue function, with consistent protective effects observed [61].

## 4. Psychiatric disorders

### 4.1. SCZ

The pathophysiology of SCZ supports the hypothesis of hypofunction of NMDARs, which could be associated with metabolites of the KYN pathway that suppress NMDARs [30] and therefore decrease neurotransmitter activities. As an antagonist of NMDARs, KYNA plays a vital role in this process [38]. The KYN pathway plays various roles in the dysregulation of the SCZ immune system. Through a meta-analysis, Cao Bing et al. reported lower levels of TRP and higher KYN/TRP ratios in SCZ patients; lower KYN was associated with SCZ patients without medication, while higher levels of KYN were observed in patients after treatment [63]. Abbas F. Almulla et al. showed an increase in IDO activity in brain tissue and peripheral serum and a decrease in KMO activity and a reduction in KYNA production in the brains of SCZ patients [64]. Impaired KMO expression and activity were also observed in postmortem brain tissues of SCZ patients [65]. Subsequent elevation of KYNA levels was suggested to have a causal link with the psychopathology of SCZ, as in the so-called "KYNA hypothesis of SCZ" [66]. Elevated levels of KYNA in developing brain tissue or CSF [67] are related to cognitive deficits [68] and psychotic symptoms [69] in SCZ. The single nucleotide polymorphism of the KMO gene is related to SCZ, further supporting the notion that the decrease in the 3-HK branch in the KP pathway may cause L-KYN to convert to KYNA, which is one of the causes of SCZ [19].

In addition to KYNA, the newly discovered 3-HK metabolite XA may play a pathogenic role in SCZ. As an endogenous agonist of the mGlu2 receptor, XA exhibits antipsychotic therapeutic effects in mice [70]. Interestingly, blood levels of XA are markedly reduced in patients affected by SCZ, regardless of the stage of the disorder or drug status, suggesting that lower levels of XA in blood may serve as a potential trait marker for SCZ [70]. Another KYNA metabolite is CA, an activator of metabotropic glutamate receptors mGlu4, which may have a therapeutic role in SCZ. However, CA did not confer any therapeutic effects in mice with mGlu4 deficiency [71]. Furthermore, CA levels were low in brain tissues from SCZ patients [71]. In particular, circulating AA levels are elevated in SCZ patients simultaneously with KYNA [70, 72] and are positively correlated with the severity of symptoms [10].

### 4.2. BD

BD is a mental illness characterized by abnormalities in the structure and function of several areas of the brain (e.g., the prefrontal cortex, anterior cingulate cortex, amygdala, and hippocampus) [73]. Although the pathomechanism of BD is not clearly understood, there is a wide range of evidence that inflammation and TRP metabolism contribute to the development of the disease [74]. The sorting nexin family is involved in the regulation of intracellular transport and signal transmission. A postmortem study of the brains of BD patients revealed that downregulation of sorting nexin 7 increased caspase-8-driven interleukin-1β production, subsequently activating the KYN pathway and resulting in neurotoxicity [75]. Another MRI study showed that the L-KYN/TRP (K/T) ratio in the blood of patients with BD was negatively correlated with amygdala volume, corpus callosum integrity, and frontal-parietal cortex thickness as quantified by MRI [76]. KYNA is considered protective in BD, but in patients with lifelong psychotic characteristics, elevated KYNA levels in the CSF are correlated with genetic mutations in KMO [77]. Therefore, the accumulation of KYNA in the brain is related to the pathophysiology of BD. Changes in melatonin secretion are also correlated with the disease state. Patients with BD with altered circadian rhythms had reduced levels of melatonin during the euphoric, manic, and depressive phases [78]. There is evidence that the level of 5-hydroxyindoleacetic acid (5HIAA), the main serotonin metabolite, is reduced in the cortex of patients with BD. Decreases in central 5-HT activity in patients with BD might cause mania and depression [79].

### 4.3. MDD

MDD is related to elevated metabolism in the 3-HK branch of the KP. The process of enhanced degradation of TRP to KYN away from serotonin production is called a "KYN shunt". The TRP degradation pathway and the KYN shunt have been implicated in MDD [80]. The shutdown of TRP metabolism from the MP to KP, moving away from serotonin production to KYN production, leads to serotonin deficiency in MDD [81]. It also results in higher levels of the neurotoxic metabolite QUINA than the neuroprotective metabolite KYNA [23]. KP activation has been clinically observed in interferon-induced depression, and LPS-induced depression-like behavior in animals has been associated with activation of the KYN pathway [80]. A clinical study showed that the ratio of KYNA to 3HK or QUINA is positively associated with hippocampal volume, and decreased hippocampal volume is correlated with impaired autobiographical memory recall in patients with MDD [23]. The levels of L-KYN, 3-HK, and KYNA are elevated in the mouse model of chronic social defeat [82]. The body produces PA to limit the formation of quinoline and inhibit suicidal behavior [82]. Changes in IDO-1 expression were analyzed in MDD patients, with higher baseline levels observed in patients than in normal controls, and the clinical efficacy of the antidepressant can be attributed at least partly to decreases in IDO1 expression. However, there are currently no reports on IDO2 expression in MDD [83]. Increased levels of IDO1 can lead to increased 3-HK production, which exerts a neurotoxic effect. The polymorphism of the IDO1 gene is related to susceptibility to cytokine-induced depression [84]. Consistent with our expectations, IDO knockout mice exhibit reduced depression-like behavior [85].

TNF, IL-6, and other inflammatory genes in peripheral blood mononuclear cells are upregulated in patients with MDD. The expression level of these genes is associated with the amygdala response to fear [82]. For a long time, evidence has linked 5-HT to the etiology of MDD. The classic antidepressant drugs, selective serotonin reuptake inhibitors (SSRIs), including sertraline and fluoxetine, exert their effects by increasing serotonin levels in the synaptic cleft [86]. Furthermore, previous studies have shown TRP depletion in the brains of MDD patients with decreased availability of the serotonin-1A receptor and the -2A receptor [87]. However, serotonin levels are increased in multiple depressive phenotypes, indicating the involvement of other mechanisms in MDD [88].

### 4.4. Therapeutic Perspectives

Targeting the TRP mechanism is an ongoing research direction to treat the psychiatric disorders mentioned above. Long-term SCZ could be controlled by blocking KYN transport to the brain or inhibiting KYNA production in the brain [19]. For example, for the treatment of SCZ, the enzyme KATII is one of the targets. The KATII inhibitor (S)-4-(ethylsulfonyl) benzoylalanine (S-ESBA) has been shown to reduce KYNA levels in the rat brain and thus enhance cognition [89]. However, the selective difference between species and the toxicity of KAT inhibitors limits their potential use [19]. Given that XA production also depends on KAT, it remains unclear whether inhibiting KAT is a worthwhile treatment option. A new direction in BD therapy is the modulation of biological rhythms. As an agonist of MT1 and MT2, agomelatine has been shown in clinical trials to be beneficial for 81% of patients with bipolar I disorder. However, it has side effects, such as mania, when combined with lithium, and other studies have shown that it has no apparent therapeutic effect [90]. Elevating endogenous melatonin levels in the brains of patients with BD is a feasible solution. Reducing KP while increasing MP may be a promising treatment prospect, such as inhibiting IDO1 with subsequent decreases in neurotoxic products. For MDD patients, two feasible treatment options include the use of KYNA competitive inhibitors, such as AV-101, and inhibiting transport of L-KYN to the brain. For example, leucine can compete with L-KYN for LAT1, thus reducing the concentration of KYNA in the brain [8]. Furthermore, previous studies have shown that 10 of the 19 amino acids can reduce L-KYN synthesis [91], suggesting that diet therapy can help in the management of mental illness. The specific pathophysiology that underlies psychiatric disorders remains unclear. Compounds targeting KP hold promise as novel treatments. Well-designed preclinical and clinical studies with KP-targeted treatment are needed to advance our understanding and the treatment of psychiatric disorders.

## 5. Acute spinal cord and brain injury

### 5.1. SCI

SCI damages the spinal cord and results in spinal cord shock. After an initial mechanical injury, damaged cells release neurotoxic glutamate [92]. Glutamate acts on the glutamate receptor AMPA, causing the death of oligodendrocytes and demyelinating nerve cells. KYNA exhibits a neuroprotective effect due to its inhibition of glutamate release, while QUINA exerts the opposite effect [93]. Previous studies have also shown that an increase in QUINA levels after SCI is related to suicidal ideation. Administration of inhibitors to reduce the production of QUINA without affecting the levels of other neuroactive substances can significantly increase the area of white matter that survives within the injured site. Administration of KYNA derivatives can improve recovery of motor function in adult male Wistar rats 1-4 weeks after SCI [93]. Furthermore, 5-HT promotes the regeneration of damaged axons [93].

### 5.2. TBI

A TBI is caused by an external force that injures the brain. Its pathogenesis involves primary and secondary injury. Inflammation leads to secondary brain damage and promotes ROS formation [94, 95]. The generation of inflammatory cytokines, such as IFN-γ and IL-6, increases the expression level of IDO. Elevated levels of IDO and QUINA are present in young rabbit models of TBI [18]. In human TBI, it has been confirmed that QUINA, L-KYN, and KYNA levels increase in the CSF. The concentration of QUINA and IDO1 expression is correlated with the worsening condition and mortality of a patient, suggesting the harmful effects of KP [15]. Acute early activation of the KYNA pathway can prevent the development of depressive symptoms after mild TBI, and QUINA in serum can potentially be a biomarker of repetitive TBI [96]. Targeting TRP metabolism can help to elucidate its pathogenic role in various emotional changes induced by TBI [97].

### 5.3. Ischemic and hemorrhagic stroke

Stroke can be classified as ischemic or hemorrhagic stroke. Ischemic stroke is caused by thrombotic or embolic occlusion of a cerebral artery. In contrast, hemorrhagic stroke (e.g., ICH) refers to the accumulation of intracerebral bleeding caused by the rupture of a penetrating artery. The pathogenesis of ischemic or hemorrhagic stroke can be divided into primary and secondary brain injury. Direct damage to ischemic stroke is due to the initial interruption of cerebral blood flow, causing a local brain infarction. The primary injury to ICH is due to increased intracranial pressure caused by physical compression of a hematoma. Secondary brain injury caused by ischemic stroke or ICH involves excitotoxicity, inflammation, and oxidative stress [98-100]. Excitotoxicity induced by TRP metabolites could be one of the mechanisms of the secondary injury. Animal experiments show that the QUINA/KYNA ratio increases in mice and gerbils, which is related to immune cell infiltration and the severity of ischemic stroke [101]. One study showed that IDO activity increases in ischemic stroke and correlates with prognosis; simultaneously, TRP levels decreased, suggesting that TRP metabolism improved [102]. The 3-HAA/AA ratio decreases when the KYNA level increases, indicating a compensatory protection mechanism against secondary brain damage [103]. Serum melatonin concentration decreases after stroke. As a neuroprotective agent, melatonin improves prognosis when administered exogenously [104]. ICH is a common and serious cerebrovascular disease with high mortality [105]. Similar to ischemic stroke, TRP metabolism may play a role in the pathophysiology of ICH. However, very few studies have investigated the effects of TRP metabolism in preclinical and clinical ICH studies [106], indicating a new direction of research.

### 5.4. Therapeutic Perspectives

Modulation of KP production is significant in the treatment of traumatic disorders, and KMO may be a new pharmacological target for the treatment of traumatic disorders [107]. Furthermore, KMO inhibitors can reduce the impact of chronic inflammation and potentially mitigate neuropathic pain and psychological disturbances after SCI [93]. TDO and KMO inhibitors also improve the prognosis of stroke patients, while IDO inhibitors do not show protection [103].

In preclinical studies, the NMDAR inhibitor memantine has improved the prognosis of ischemic stroke [108]. Furthermore, as an endogenous ligand for NMDAR, KYNA is a promising drug candidate with fewer CNS side effects. For example, N-(2-N,N-dimethylaminoethyl)-4-oxo-1H-quinoline-2-carboxamide hydrochloride, a KYNA analog, has shown therapeutic efficacy [109].

5-HT and melatonin are also drug candidates for drugs for traumatic disorders. Potential treatment strategies for SCI include the regulation of neuroinflammation and the transplantation of mesenchymal stem cells and neural precursors to create a preregeneration environment and increase the preregeneration capacity of damaged neurons. 5-HT can promote the regeneration of damaged axons. Increased 5-HT axon density at the injured site is a good indicator of improved functional recovery [110]. Furthermore, melatonin reduces oxidative stress and inflammation by activating the signaling pathway of nuclear factor erythroid 2-related factor 2 and alleviates secondary damage to TBI [94]. Oral melatonin can reduce the severity of stroke patients and improve cognitive decline [104].

## 6. Autoimmune diseases

### 6.1. MS

MS is an autoimmune-mediated disorder characterized by the formation of sclerotic plaques, inflammation, and demyelination [111]. There is an apparent KP imbalance in MS, although the exact role of KP is not fully clear. Preclinical studies have shown that IDO1 activity decreases in the brain and spinal cord of mice with experimental autoimmune encephalomyelitis (EAE), leading to increased Th1 and Th17-cell activity and reduced Treg cell activity, worsening the severity of the disease [112, 113]. Another study showed that after increasing IDO1 activity, 3-HAA in KP increased, resulting in an immunosuppressive effect [114]. Furthermore, the results of an in vitro study indicated that QUINA can alter oligodendrocytes, thus expanding its pathogenic effects in MS [22].

Clinical trials have shown that enhancing IDO1 suppresses immune responses, suggesting that IDO1 exerts anti-inflammatory and neuroprotective effects [44]. The CSF level of TRP decreases in MS patients [22]. The accumulation of QUINA is related to the severity of the disease [111]. The level of KYNA in the CSF of MS patients is significantly reduced in remission. In contrast, KYNA levels in plasma and CSF increase during acute relapse and decrease during chronic remission [22]. These findings indicate that KYNA may be involved in the relapse-remission phase of the disease. In the CSF, the PA level decreases while the QUINA level increases, which implies that KP metabolism switches to the neurotoxic pathway [111]. 5-HT may play a pathogenic role in MS as its expression level in the CSF is decreased in MS patients [115]. NAS, the 5-HT metabolite, interacts directly with IDO1 and functions as a positive allosteric modulator to elevate the level of L-KYN [116]. It also activates AhR, thus exerting anti-inflammatory and immunoregulatory effects [116].

### 6.2. Therapeutic Perspectives

In summary, TRP metabolites and enzymes play a significant role in the pathogenesis of MS. The data suggest that by regulating the production or activity of these enzymes or metabolites or using synthetic analogs, MS can be managed or treated. Previous studies suggest that increasing IDO activity and inhibiting the production of downstream catabolic products hinder the progression of EAE and may be a new therapeutic strategy [117]. The KYNA analog laquinimod has been used as a disease-modifying therapy to treat MS in multiple stages [118]. In addition to anti-inflammatory and immune regulation, laquinimod protects astrocytes and oligodendrocytes [119]. It also reduces axon damage and promotes remyelination [120]. However, due to its severe adverse effects, it cannot be used currently as a drug in clinical practice [120]. Therefore, more studies are needed to investigate drug candidates with better and superior curative effects with fewer side effects [44].

Increasing the level of 5-HT can provide therapeutic benefits. Phenelzine can increase the content of 5-HT between axons and has also been shown to improve behavioral performance in a mouse model of EAE [121]. However, clinical trials have shown that fluoxetine, another drug that can increase the 5-HT concentration in the brain, did not benefit MS patients significantly [122]. The reason may be the small sample size, the baseline imbalance, or confounding bias [115]. These two contrasting results suggest that alternative mechanisms that regulate 5-HT may need to be investigated.

## 7. Neurodegeneration

### 7.1. AD

AD is the most common form of dementia, with continuous cognitive impairment and progressive memory loss [123]. Deposition of misfolded proteins such as amyloid beta-protein (Aβ) and hyperphosphorylated tau, loss of synaptic transmission, and neuroinflammation are characteristics of AD [124]. Tryptophan metabolites, QUINA and KYNA, together with other intermediates, show elevated concentrations associated with Aβ and tau in the CSF of AD patients, which are neurodegeneration biomarkers, suggesting that TRP is closely related to the core pathology of AD [125]. One study showed that CSF levels of Aβ40 and Aβ42 correlated with those of K/T, KYNA, L-KYN, QUINA, and AA in AD patients with a high neocortical Aβ load [125]. Furthermore, high levels of QUINA are associated with neuronal damage in AD [126]. An increased K/T ratio in serum and CSF indicates that TRP metabolism is enhanced, and IDO activation increases in AD [5].

KYNA activation increases NAD^+^ production, promotes DNA repair, strengthens genomic signaling, and generates more energy [127]. Therefore, it plays an important role in antioxidant defense and protection against AD [128]. Neurotoxic QUINA, mainly produced by activated microglia, can induce tau phosphorylation [129]. Interestingly, IDO-1 is colocalized with extracellular Aβ plaques [130]. KYNA levels are elevated in the CSF [131] but decreased in serum [132]. A recent study demonstrated that 3-HK and 3-HAA increase Cu^++^-induced neurotoxicity in rat astrocyte cultures [133]. However, the mechanism behind the neuronal death induced by TRP metabolites is not fully understood. Melatonin is a branch of TRP metabolism. As an antioxidant, free radical scavenger, protein aggregation inhibitor, anti-inflammatory agent, and regulator of various enzymes, melatonin exerts a neuroprotective role in delaying the development of AD [134]. The available evidence suggests that TRP metabolism is involved in the pathogenesis of AD. Therefore, targeting TRP metabolism will help to elucidate the pathomechanism of AD.

### 7.2. PD

PD is another common age-related neurodegenerative disease. Degeneration of dopaminergic neurons in the substantia nigra pars compacta produces characteristic motor symptoms [5]. Alpha-synuclein and dopamine metabolites in the CSF are considered biomarkers of PD [135]. Serum levels of TRP, L-KYN, and KYNA are lower in PD patients than in controls [136]. The KP of TRP catabolism can regulate inflammatory and neurotoxic processes in PD. The K/T ratio and 3-HK levels in the putamen, frontal cortex, and hippocampus of PD patients is significantly increased, indicating an increase in IDO/TDO activity in PD patients [137]. The KYNA level is decreased in the striatum and CSF of patients with PD and correlated with an increase in excitotoxicity, while the QUINA level is increased in the striatum and cortex [138]. QUINA and 3-HK are involved in the pathogenesis of PD. Related underlying mechanisms include NMDAR activation, ROS production [139], lipid peroxidation, and increased levels of nitric oxide synthase [136]. There is a significant correlation between increased neopterin levels and the K/T ratio in the serum and CSF of patients with PD [137], with high cell-mediated immunity (e.g., CD4^+^ T cells and CD8^+^ T cells) in patients with advanced PD.

### 7.3. ALS

ALS is a progressive neurodegenerative disease characterized by selective death of motor neurons in the cerebral cortex, brainstem, and spinal cord, leading to atrophy of voluntary muscles, weakness, paralysis, and premature death [140]. In the brain, the level of TRP regulates serotonin production. Depression is related to decreased serotonin levels caused by TRP depletion [141] and sleep disturbances caused by decreased melatonin, both of which are symptoms in patients with ALS. The most commonly measured KPMs are L-KYN, TRP, KYNA, XA, and AA, and their levels are stable in ALS [142]. Astrocytosis is a feature of neuroinflammation in ALS. Activated microglia and activated KP are present in the motor cortex of patients with ALS, and the levels of TRP, L-KYN and QUINA in the CSF and serum are increased [143]. When the disease worsens, the high KYNA level is proportional to the severity and is neuroprotective. One study showed no significant differences in KYNA levels in serum or CSF between patients and control subjects [142]. However, the level of KYNA in CSF was higher in patients with severe clinical conditions than in control subjects, and the KYNA concentrations in serum and CSF are not related [142]. CSF levels of QUINA and IDO increased significantly in patients with ALS [6]. Activated voluminous microglia, reactive astrocytes, infiltrating macrophages, and T cells are present in the brain and spinal cord of ALS patients, suggesting that these inflammatory cells play a role in the pathogenesis of ALS [144], particularly glutamate excitotoxicity, oxidative stress, mitochondrial metabolic dysfunction, neuroinflammation, protein aggregation, and autophagy [144]. These data suggest that QUINA contributes to the pathophysiology of ALS.

### 7.4. HD

HD is an autosomal dominant neurogenetic disorder. Its pathogenesis involves the repeat expansion of CAG in the first exon of the huntingtin gene (HTT), which encodes a polyglutamine stretch in the HTT protein [145]. Preclinical studies have shown that melatonin is significantly increased in presymptomatic HD sheep [146], and a self-protective response to the toxicity of the mutant huntingtin protein could be the cause [146]. Experiments with human samples and animal genetic models of HD have demonstrated that neuroactive metabolites in KP play a role in the development of HD [147]. Clinical studies have shown that the level of 3-HK increases in the brains of patients [148], which has a toxic effect on the striatum, thus increasing the production of toxic QUINA. Increased QUINA causes a certain degree of excitotoxicity or oxidative stress, leading to striatal neuronal dysfunction [6]. A clinical study showed that the K/T ratio in the CSF is higher in HD patients than in control subjects at baseline and after TRP depletion, which can be caused by eating an amino acid mixture containing all essential amino acids except TRP [149]. The results suggested an increase in IDO activity in the HD brain.

Furthermore, HD patients exhibit abnormal TRP metabolism and increased oxidative stress. These factors contribute to continued brain dysfunction. Plasma levels of KYNA, 3-HK, and 3-HAA and KAT activity are decreased in HD [149]. Inhibition of KMO activity reduces QUINA production in HD brain tissues [77], suggesting that the KP inhibitor can reduce huntingtin-induced abnormal cytotoxicity.

**Table 2:** Changes in the kynurenine pathway in psychiatric disorders, acute brain and spinal cord disorders, and multiple sclerosis.

Brain (CSF)	SCZ	BD	MDD	TBI	SCI	Stroke	MS
TRP	↓ [70]	↓ [162]	↓ [163]	N [15]	-	↓ [103]	↓ [22]
QUINA	↓ [70]	N [162]	↑ [23]	↑ [15]	↑ [93]	↑ [103]	N or ↑ ^#^
KYNA	↑ [65]	↓ [162]	↓ [163]	↑ [15]	-	↓ [102]	↑ or ↓*
K/T	↑ [65]	N [162]	↑ [163]	↑ [15]	↑ [164]	↑ [165]	↑ [44]
Melatonin	↓ [166]	↓ [167]	↓ [168]	↓ [18]	↓ [169]	↓ [104]	↓ [170]
IDO	↑ [65]	↑ [171]	↑ [172]	↑ [18]	↑ [107]	↑ [102]	↑ [173]
5-HT	↑ [174]	↓ [79]	↑ [88]	↓ [18]	↓ [110]	↓ [175]	↓ [115]
L-KYN	↑ [63]	N [162]	N [163]	↑ [15]	↑ [93]	↑ [176]	↑ [116]
3-HK	↑ [8]	↑ [177]	↑ [82]	↑ [96]	↑ [93]	↑ [178]	↑ [44]
3-HAA	↑ [70]	-	N [179]	N [15]	↑ [93]	↓ [161]	↑ [44]

↑: increase; ↓: decrease; N: nonsignificant difference; - uncertainty ^#^ There was no difference in the CSF level of QUINA between MS patients and normal subjects, but QUINA was increased in patients with relapsing-remitting MS (RRMS) in the relapse stage [180]. *KYNA increases during acute relapse and decreases during chronic remission [22]. Abbreviations: 3-HAA: 3-hydroxyanthrenillc acid; 3-HK: 3-hydroxykynurenine; 5-HT: 5-hydroxy tryptophan; BD: bipolar disorder; CSF: cerebrospinal fluid; IDO: indoleamine 2,3-dioxygenase; K/T: L-KYN/TRP; KYNA: kynurenic acid; L-KYN: L-kynurenine; MDD: major depressive disorder; MS: multiple sclerosis; QUINA: quinolinic acid; SCI: spinal cord injury; SCZ: schizophrenia; TBI: traumatic brain injury; TRP: L-tryptophan.

### 7.5. Therapeutic Perspectives

In general, inhibitors of the key enzymes in KP are believed to be therapeutic candidates for neuro-degenerative diseases. KMO is located at the point of branch of QUINA and KYNA production. Therefore, drugs that specifically inhibit KMO activity, such as JM6 and Ro61-8048, can change metabolism to increase KYNA levels and reduce extracellular glutamate in the brain, providing neuroprotection [150]. Metabolites of the KP of TRP degradation, such as QUINA, induce cerebral oxidative stress and trigger the inflammatory response in several neurodegenerative diseases. As a phytochemical with antioxidant and anti-inflammatory activities, phenolic compounds can treat neurodegenerative diseases [151]. With similar biological properties, melatonin could also be a candidate drug [134].

Targeting TRP metabolism can help in the development of disease-modifying therapies for AD. The serotonin receptor (a G protein-coupled receptor) 5-HT4 and 5-HT6 receptors are new potential drug targets [152]. One of the main effects of 5-HT6R activation is to reduce cholinergic transmission. Therefore, receptor antagonists can increase ACh production and stimulate cholinergic transmission. KYNA functions on the Ach receptor as a noncompetitive antagonist since the Ach concentration is decreased in AD [33]. The drugs available today exert only a symptomatic effect. Therefore, acetylcholine-sterase and butyrylcholinesterase inhibitors remain FDA-approved drugs for the treatment of AD [153], including rivastigmine and galantamine [154]. TRP metabolites have tremendous therapeutic potential in AD, KYNA, and 5-HIAA and can reduce the accumulation of Aβ in the brain [155]. Exploring 5-HIAA analogs or precursors using KMO combined with KYNA may become a new research direction.

Although dopaminergic agonists (e.g., ropinirole, pramipexole, rotigotine), L-DOPA, and carbidopa are widely used to treat PD, they provide symptomatic relief but not a cure. Furthermore, the pathomechanism of the progression of metabolite-mediated disease of these drugs remains elusive [156]. However, inhibition of KMOs such as Ro 61-8048 can increase KYNA levels in PD brain tissue [157], which could be a potential drug candidate for the treatment of PD [156]. Treatment targeting TRP includes probenecid, L-KYN + probenecid, and nicotinylalanine + L-KYN, which have been shown to increase KYNA levels in the brain and delay the neurodegeneration process of PD under in vitro and in vivo conditions [157].

There are three possible ways to treat ALS. The first is inhibition of the enzyme in KP. The second target is the precursors of NAD or KYNA, such as nicotinamide mononucleotide, which have been used to treat aging and neurodegeneration [158]. The third is synthetic drugs with high bioavailability and affinity for excitatory receptor binding sites [119]. Although Riluzole was the first drug approved by the FDA to treat ALS in 1995 [144], there is still a lack of effective treatments for ALS today. Therefore, joint efforts of basic scientists and physician scientists in preclinical and clinical studies are needed.

Reducing presynaptic dopamine or blocking the D2 dopamine receptor is the current therapeutic strategy for treating chorea. Tetrabenazine is the only approved drug for treating chorea caused by HD [159]. IDO1 could be a new therapeutic target for HD by regulating inflammatory processes and neurodegeneration and inducing depressive symptoms in HD [145]. Similar to other neurodegenerative diseases, KMO inhibitors could also be a new therapeutic target for HD [160].

**Table 3:** Changes in the kynurenine pathway in neurodegenerative diseases.

		AD	PD	ALS	HD
Brain	TRP	↓ [125]	↓ [181]	↑ [143]	↓ [182]
	QUIN	↑ [29]	↑ [138]	↑ [142]	↑ [6]
	KYNA	↑ [29]	↓ [138]	N [142]	N [145]
	K/T	↑ [5]	↑ [137]	↑ [142]	↑ [182]
	Melatonin	↓ [134]	↓ [134]	↓ [134]	↓ [146]
	IDO	↑ [183]	↑ [137]	↑ [184]	↑ [145]
	3-HK	↑ [130]	↑ [137]	↑ [142]	↑ [148]
Serum	TRP	↓ [125]	↓ [50]	↑ [143]	↓ [182]
	QUINA	↑ [29]	↑ [137]	↑ [142]	↑ [145]
	KYNA	↓ [132]	↓ [136]	↓ [142]	↓ [149]
	K/T	↑ [5]	↑ [185]	↑ [142]	↑ [182]
	3-HK	↓ [185]	N [185]	-	↓ [149]
	3- HAA	↑ [133]	↓ [138]	-	↓ [149]
	L-KYN	↑ [125]	↑ [186]	↑ [143]	↑ [6]

↑: increase; ↓: decrease; N: nonsignificant difference; -: uncertainty. Abbreviations: 3-HAA: 3-hydroxyanthrenillc acid; 3-HK: 3-hydroxykynurenine; 5-HT: 5-hydroxy tryptophan; AD: Alzheimer’s disease; ALS: amyotrophic lateral sclerosis; HD: Huntington’s disease; IDO: indoleamine 2,3-dioxygenase; K/T: L-KYN/TRP; KYNA: kynurenic acid; L-KYN: L-kynurenine; PD: Parkinson's disease; QUINA: quinolinic acid; TRP: L-tryptophan.

## 8. Conclusion

In this review, we summarized and discussed the latest research on the TRP metabolic pathway and its primary metabolites, elucidated its pathogenic role in 12 types of CNS disease, and noted that KP-targeted drugs have therapeutic potential to treat CNS diseases. When the CNS is injured, the metabolic pathway of TRP switches to KP. Inhibition of MP reduces the release of melatonin accordingly (Tables 2 and 3). L-KYN has two metabolic pathways, the neurotoxic QUINA pathway and the neuroprotective KYNA pathway. In general, the activities of the metabolites of these two pathways antagonize each other. The QUINA pathway is overactivated in various CNS diseases, whereas the KYNA branch is inhibited. Therefore, therapeutic strategies that inhibit the QUINA pathway and promote the KYNA pathway can modulate TRP metabolism and improve the histological and functional outcomes of CNS diseases. Research on specific enzyme inhibitors in the KP pathway has much room for exploration, and we summarize relevant studies related to CNS diseases mainly in 2015-2022 in Table 4.

Meanwhile, MP metabolites also have tremendous therapeutic potential for CNS diseases, and it is essential to investigate treatment options and administration routes related to 5-HT and melatonin. Continuous advances in preclinical and clinical research in this area can bring hope to the fight against various CNS diseases. Above all, relevant translational research is essential to identify therapeutic strategies for the treatment of traumatic conditions. Furthermore, exploring therapeutic options that simultaneously regulate KP and MP can maximize benefits, supporting the potential of targeting TRP metabolism.

## 9. Search strategy and selection criteria

We used major scientific databases, such as PubMed, Web of Science, Home Springer, and ScienceDirect, for literature searches, mainly from 2015. We searched through different combinations of the following keywords: TRP metabolism or TRP or kynurenine pathway or methoxyindole pathway, KYNA or QUINA or 3-HK or 3-HAA or IDO or 5-HT or melatonin, CNS disease or neurodegenerative disease or mental disorder or CNS or brain injury, biomarkers, targets, or treatment. References to related articles, bibliographies of articles, and relevant book chapters were also included in the scope of our literature search. We only selected published articles in English and focused mainly on studies published since 2015. A total of 285 articles were reviewed, 90 articles were excluded, and we finally discussed 195 articles. The articles were shortlisted according to the title and abstract, and we chose articles that described TRP metabolism in CNS diseases. We also discuss relevant biomarkers and therapeutic strategies.

**Table 4:** Inhibitors of KP enzymes tested in preclinical models of brain disorders.

Inhibitors	Names	Structural Formula	Indications	Comments
IDO inhibitors	1-methyl-l-tryptophan (L-1-MT)	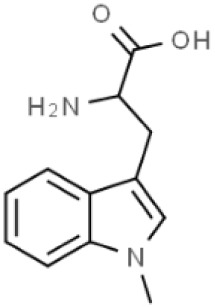	Depression/Preclinical [187]	Poor pharmacokinetics [188]; Competitive inhibitor [189].
	Coptisine	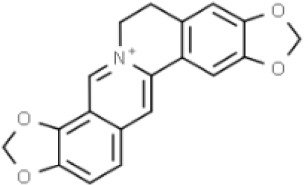	AD/Preclinical	Noncompetitive inhibitor. Potent inhibitor of recombinant human IDO [183].
TDO inhibitors	68OC91	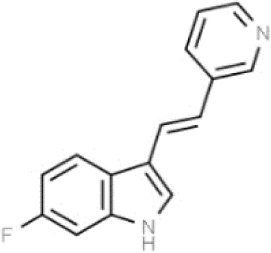	Cognitive deficits and anxiety/Preclinical	Potent inhibitor without influencing 5-HT reuptake. Poor solubility and bioavailability [190].
	Allopurinol	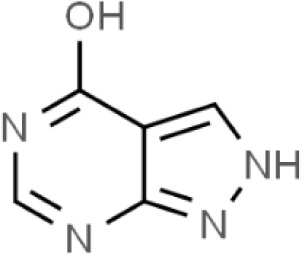	Stress/Preclinical	Uncertain about structure and lack of clinical trials [190].
	(S)-4-(ethylsulfonyl)benzoyl Alanine (S-ESBA)	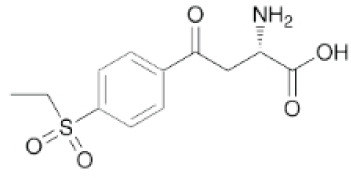	Neurodegenerative and Cognitive disorders/Preclinical [191]	KATII selective. Lowers the KYNA level in the rat brain [192]; Low activity against human KATII [19].
KATII inhibitors	BFF-122	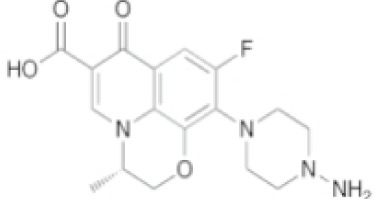	Neurodegenerative and Cognitive disorders/Preclinical [191]	Irreversible KATII inhibitor. Irreversible PLP deactivators [191].
	PF-04859989	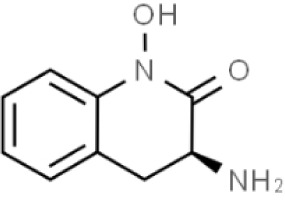	Neurodegenerative and Cognitive disorders/Preclinical [191]	Strong therapeutic effect in the rat. BBB permeable [192].
	ZINC35466084	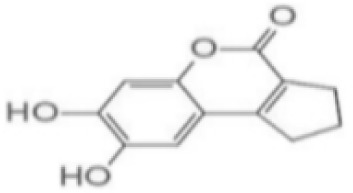	Experimental/laboratory phase	Potent inhibitor [193].
	JN-01 and JN-02	No information	Experimental/laboratory phase	Potent inhibitor [194]
KMO inhibitors	UPF-648	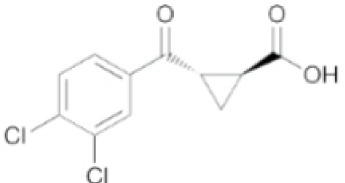	Neuropathy/Preclinical [107]	Acts as an effector molecule of KMO. Reduction of flavin by NADPH. Generating the cytotoxic H_2_O_2_ [195].
	Ro 61-8048	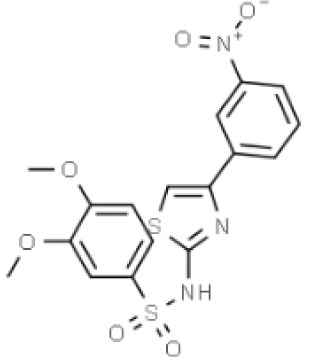	Neurodegenerative or neurologic disorders/Preclinical [196]	Competitive inhibitor [197]; Poor penetration of BBB in rats [195].
	CHDI-340246	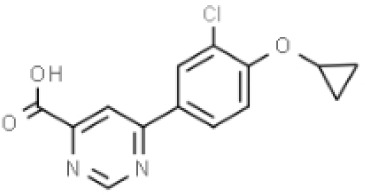	HD/Preclinical	No therapeutic efficacy in a mouse model of HD [198].

Abbreviations: 5-HT, 5-hydroxytryptophan; AD, Alzheimer’s disease; BBB: blood-brain barrier; CNS, central nervous system; HD, Huntington disease; IDO, indoleamine-2,3-dioxygenase; KAT, kynurenine aminotransferase I-III; KMO, kynurenine 3-monooxygenase; KP, kynurenine pathway; KYNA, kynurenic acid; PPP, pyridoxal-51-phosphate; TDO, tryptophan-2,3-dioxygenase.

## References

[1] Comai S, Bertazzo A, Brughera M, Crotti S (2020). Tryptophan in health and disease. Adv Clin Chem, 95:165-218. 32122523 10.1016/bs.acc.2019.08.005

[2] Generoso JS, Giridharan VV, Lee J, Macedo D, Barichello T (2021). The role of the microbiota-gut-brain axis in neuropsychiatric disorders. Braz J Psychiatry, 43:293-305. 32667590 10.1590/1516-4446-2020-0987 PMC8136391

[3] Srivastava S (2016). Emerging therapeutic roles for NAD(+) metabolism in mitochondrial and age-related disorders. Clin Transl Med, 5:25. 27465020 10.1186/s40169-016-0104-7 PMC4963347

[4] Hertelendy P, Toldi J, Fülöp F, Vécsei L (2018). Ischemic Stroke and Kynurenines: Medicinal Chemistry Aspects. Curr Med Chem, 25:5945-5957. 29532751 10.2174/0929867325666180313113411

[5] Bohár Z, Toldi J, Fülöp F, Vécsei L (2015). Changing the face of kynurenines and neurotoxicity: therapeutic considerations. Int J Mol Sci, 16:9772-9793. 25938971 10.3390/ijms16059772 PMC4463617

[6] Török N, Tanaka M, Vécsei L (2020). Searching for Peripheral Biomarkers in Neurodegenerative Diseases: The Tryptophan-Kynurenine Metabolic Pathway. Int J Mol Sci, 21:9338 33302404 10.3390/ijms21249338 PMC7762583

[7] Albrecht J, Zielińska M (2019). Exchange-mode glutamine transport across CNS cell membranes. Neuropharmacology, 161:107560. 30853601 10.1016/j.neuropharm.2019.03.003

[8] Savitz J (2020). The kynurenine pathway: a finger in every pie. Mol Psychiatry, 25:131-147. 30980044 10.1038/s41380-019-0414-4 PMC6790159

[9] Pawlowski T, Pawlak D, Inglot M, Zalewska M, Marciniak D, Bugajska J, et al. (2021). The role of anthranilic acid in the increase of depressive symptoms and major depressive disorder during treatment for hepatitis C with pegylated interferon-α2a and oral ribavirin. J Psychiatry Neurosci, 46:E166-e175. 33464780 10.1503/jpn.190139 PMC7955854

[10] Steiner J, Dobrowolny H, Guest PC, Bernstein HG, Fuchs D, Roeser J, et al. (2022). Gender-specific elevation of plasma anthranilic acid in schizophrenia: Protection against glutamatergic hypofunction? Schizophr Res, 243:483-485. 35151533 10.1016/j.schres.2022.01.048

[11] Zhai L, Ladomersky E, Lenzen A, Nguyen B, Patel R, Lauing KL, et al. (2018). IDO1 in cancer: a Gemini of immune checkpoints. Cell Mol Immunol, 15:447-457. 29375124 10.1038/cmi.2017.143 PMC6068130

[12] Barone P (2019). The 'Yin' and the 'Yang' of the kynurenine pathway: excitotoxicity and neuroprotection imbalance in stress-induced disorders. Behav Pharmacol, 30:163-186. 30844963 10.1097/FBP.0000000000000477

[13] Chiappelli J, Notarangelo FM, Pocivavsek A, Thomas MAR, Rowland LM, Schwarcz R, et al. (2018). Influence of plasma cytokines on kynurenine and kynurenic acid in schizophrenia. Neuropsychopharmacology, 43:1675-1680. 29520060 10.1038/s41386-018-0038-4 PMC6006321

[14] Songtachalert T, Roomruangwong C, Carvalho AF, Bourin M, Maes M (2018). Anxiety Disorders: Sex Differences in Serotonin and Tryptophan Metabolism. Curr Top Med Chem, 18:1704-1715. 30430940 10.2174/1568026618666181115093136

[15] Yan EB, Frugier T, Lim CK, Heng B, Sundaram G, Tan M, et al. (2015). Activation of the kynurenine pathway and increased production of the excitotoxin quinolinic acid following traumatic brain injury in humans. J Neuroinflammation, 12:110. 26025142 10.1186/s12974-015-0328-2 PMC4457980

[16] Lovelace MD, Varney B, Sundaram G, Franco NF, Ng ML, Pai S, et al. (2016). Current Evidence for a Role of the Kynurenine Pathway of Tryptophan Metabolism in Multiple Sclerosis. Front Immunol, 7:246. 27540379 10.3389/fimmu.2016.00246 PMC4972824

[17] Mondanelli G, Iacono A, Carvalho A, Orabona C, Volpi C, Pallotta MT, et al. (2019). Amino acid metabolism as drug target in autoimmune diseases. Autoimmun Rev, 18:334-348. 30797943 10.1016/j.autrev.2019.02.004

[18] Zhang Z, Rasmussen L, Saraswati M, Koehler RC, Robertson C, Kannan S (2019). Traumatic Injury Leads to Inflammation and Altered Tryptophan Metabolism in the Juvenile Rabbit Brain. J Neurotrauma, 26:74-86. 10.1089/neu.2017.5450 PMC12430859 30019623

[19] Platten M, Nollen EAA, Röhrig UF, Fallarino F, Opitz CA (2019). Tryptophan metabolism as a common therapeutic target in cancer, neurodegeneration and beyond. Nat Rev Drug Discov, 18:379-401. 30760888 10.1038/s41573-019-0016-5

[20] Merlo LMF, DuHadaway JB, Montgomery JD, Peng WD, Murray PJ, Prendergast GC, et al. (2020). Differential Roles of IDO1 and IDO2 in T and B Cell Inflammatory Immune Responses. Front Immunol, 11:1861. 32973768 10.3389/fimmu.2020.01861 PMC7461966

[21] Li Y, Hu N, Yang D, Oxenkrug G, Yang Q (2017). Regulating the balance between the kynurenine and serotonin pathways of tryptophan metabolism. FEBS J, 284:948-966. 28118532 10.1111/febs.14026

[22] Vécsei L, Szalárdy L, Fülöp F, Toldi J (2013). Kynurenines in the CNS: recent advances and new questions. Nat Rev Drug Discov, 12:64-82. 23237916 10.1038/nrd3793

[23] Young KD, Drevets WC, Dantzer R, Teague TK, Bodurka J, Savitz J (2016). Kynurenine pathway metabolites are associated with hippocampal activity during autobiographical memory recall in patients with depression. Brain Behav Immun, 56:335-342. 27091600 10.1016/j.bbi.2016.04.007 PMC4917447

[24] Meier TB, Savitz J (2022). The Kynurenine Pathway in Traumatic Brain Injury: Implications for Psychiatric Outcomes. Biol Psychiatry, 91:449-458. 34266671 10.1016/j.biopsych.2021.05.021 PMC8630076

[25] Kado S, Chang WLW, Chi AN, Wolny M, Shepherd DM, Vogel CFA (2017). Aryl hydrocarbon receptor signaling modifies Toll-like receptor-regulated responses in human dendritic cells. Arch Toxicol, 91:2209-2221. 27783115 10.1007/s00204-016-1880-y PMC5400689

[26] Campesato LF, Budhu S, Tchaicha J, Weng CH, Gigoux M, Cohen IJ, et al. (2020). Blockade of the AHR restricts a Treg-macrophage suppressive axis induced by L-Kynurenine. Nat Commun, 11:4011. 32782249 10.1038/s41467-020-17750-z PMC7419300

[27] Kruse JL, Cho JH, Olmstead R, Hwang L, Faull K, Eisenberger NI, et al. (2019). Kynurenine metabolism and inflammation-induced depressed mood: A human experimental study. Psychoneuroendocrinology, 109:104371. 31325802 10.1016/j.psyneuen.2019.104371 PMC6842695

[28] Zakhary G, Sherchan P, Li Q, Tang J, Zhang JH (2020). Modification of kynurenine pathway via inhibition of kynurenine hydroxylase attenuates surgical brain injury complications in a male rat model. J Neurosci Res, 98:155-167. 31257634 10.1002/jnr.24489 PMC6854312

[29] Maddison DC, Giorgini F (2015). The kynurenine pathway and neurodegenerative disease. Semin Cell Dev Biol, 40:134-141. 25773161 10.1016/j.semcdb.2015.03.002

[30] Schwarcz R, Bruno JP, Muchowski PJ, Wu HQ (2012). Kynurenines in the mammalian brain: when physiology meets pathology. Nat Rev Neurosci, 13:465-477. 22678511 10.1038/nrn3257 PMC3681811

[31] Ramos-Chávez LA, Lugo Huitrón R, González Esquivel D, Pineda B, Ríos C, Silva-Adaya D, et al. (2018). Relevance of Alternative Routes of Kynurenic Acid Production in the Brain. Oxid Med Cell Longev, 2018:5272741. 29977455 10.1155/2018/5272741 PMC5994304

[32] Tóth F, Cseh EK, Vécsei L (2021). Natural Molecules and Neuroprotection: Kynurenic Acid, Pantethine and α-Lipoic Acid. Int J Mol Sci, 22:403. 33401674 10.3390/ijms22010403 PMC7795784

[33] Sharma R, Razdan K, Bansal Y, Kuhad A (2018). Rollercoaster ride of kynurenines: steering the wheel towards neuroprotection in Alzheimer's disease. Expert Opin Ther Targets, 22:849-867. 30223691 10.1080/14728222.2018.1524877

[34] Cervenka I, Agudelo LZ, Ruas JL (2017). Kynurenines: Tryptophan's metabolites in exercise, inflammation, and mental health. Science, 357:eaaf9794. 28751584 10.1126/science.aaf9794

[35] Tiszlavicz Z, Németh B, Fülöp F, Vécsei L, Tápai K, Ocsovszky I, et al. (2011). Different inhibitory effects of kynurenic acid and a novel kynurenic acid analogue on tumour necrosis factor-α (TNF-α) production by mononuclear cells, HMGB1 production by monocytes and HNP1-3 secretion by neutrophils. Naunyn Schmiedebergs Arch Pharmacol, 383:447-455. 21336543 10.1007/s00210-011-0605-2

[36] Fallarini S, Magliulo L, Paoletti T, de Lalla C, Lombardi G (2010). Expression of functional GPR35 in human iNKT cells. Biochem Biophys Res Commun, 398:420-425. 20599711 10.1016/j.bbrc.2010.06.091

[37] DiNatale BC, Murray IA, Schroeder JC, Flaveny CA, Lahoti TS, Laurenzana EM, et al. (2010). Kynurenic acid is a potent endogenous aryl hydrocarbon receptor ligand that synergistically induces interleukin-6 in the presence of inflammatory signaling. Toxicol Sci, 115:89-97. 20106948 10.1093/toxsci/kfq024 PMC2855350

[38] Phenis D, Vunck SA, Valentini V, Arias H, Schwarcz R, Bruno JP (2020). Activation of alpha7 nicotinic and NMDA receptors is necessary for performance in a working memory task. Psychopharmacology (Berl), 237:1723-1735. 32162104 10.1007/s00213-020-05495-y PMC7313359

[39] Favennec M, Hennart B, Caiazzo R, Leloire A, Yengo L, Verbanck M, et al. (2015). The kynurenine pathway is activated in human obesity and shifted toward kynurenine monooxygenase activation. Obesity (Silver Spring), 23:2066-2074. 26347385 10.1002/oby.21199

[40] Parrott JM, O'Connor JC (2015). Kynurenine 3-Monooxygenase: An Influential Mediator of Neuropathology. Front Psychiatry, 6:116. 26347662 10.3389/fpsyt.2015.00116 PMC4542134

[41] Guillemin GJ, Kerr SJ, Smythe GA, Smith DG, Kapoor V, Armati PJ, et al. (2001). Kynurenine pathway metabolism in human astrocytes: a paradox for neuronal protection. J Neurochem, 78:842-853. 11520905 10.1046/j.1471-4159.2001.00498.x

[42] Guillemin GJ, Cullen KM, Lim CK, Smythe GA, Garner B, Kapoor V, et al. (2007). Characterization of the kynurenine pathway in human neurons. J Neurosci, 27:12884-12892. 18032661 10.1523/JNEUROSCI.4101-07.2007 PMC6673280

[43] Schwarcz R, Guidetti P, Sathyasaikumar KV, Muchowski PJ (2010). Of mice, rats and men: Revisiting the quinolinic acid hypothesis of Huntington's disease. Prog Neurobiol, 90:230-245. 19394403 10.1016/j.pneurobio.2009.04.005 PMC2829333

[44] Biernacki T, Sandi D, Bencsik K, Vécsei L (2020). Kynurenines in the Pathogenesis of Multiple Sclerosis: Therapeutic Perspectives. Cells, 9:1564. 32604956 10.3390/cells9061564 PMC7349747

[45] Hansen KB, Yi F, Perszyk RE, Menniti FS, Traynelis SF (2017). NMDA Receptors in the Central Nervous System. Methods Mol Biol, 1677:1-80. 28986865 10.1007/978-1-4939-7321-7_1 PMC7325486

[46] Sugumar M, Sevanan M, Sekar S (2019). Neuroprotective effect of naringenin against MPTP-induced oxidative stress. Int J Neurosci, 129:534-539. 30433834 10.1080/00207454.2018.1545772

[47] Guillemin GJ (2012). Quinolinic acid, the inescapable neurotoxin. FEBS J, 279:1356-1365. 22248144 10.1111/j.1742-4658.2012.08485.x

[48] Braidy N, Grant R, Adams S, Brew BJ, Guillemin GJ (2009). Mechanism for quinolinic acid cytotoxicity in human astrocytes and neurons. Neurotox Res, 16:77-86. 19526301 10.1007/s12640-009-9051-z

[49] Fazio F, Lionetto L, Curto M, Iacovelli L, Copeland CS, Neale SA, et al. (2017). Cinnabarinic acid and xanthurenic acid: Two kynurenine metabolites that interact with metabotropic glutamate receptors. Neuropharmacology, 112:365-372. 27342123 10.1016/j.neuropharm.2016.06.020

[50] Lowe MM, Mold JE, Kanwar B, Huang Y, Louie A, Pollastri MP, et al. (2014). Identification of cinnabarinic acid as a novel endogenous aryl hydrocarbon receptor ligand that drives IL-22 production. PLoS One, 9:e87877. 24498387 10.1371/journal.pone.0087877 PMC3912126

[51] Taleb O, Maammar M, Brumaru D, Bourguignon JJ, Schmitt M, Klein C, et al. (2012). Xanthurenic acid binds to neuronal G-protein-coupled receptors that secondarily activate cationic channels in the cell line NCB-20. PLoS One, 7:e48553. 23139790 10.1371/journal.pone.0048553 PMC3491036

[52] Zuwała-Jagiello J, Pazgan-Simon M, Simon K, Warwas M (2012). Picolinic acid in patients with chronic hepatitis C infection: a preliminary report. Mediators Inflamm, 2012:762863. 22701277 10.1155/2012/762863 PMC3368595

[53] Yoo JM, Lee BD, Sok DE, Ma JY, Kim MR (2017). Neuroprotective action of N-acetyl serotonin in oxidative stress-induced apoptosis through the activation of both TrkB/CREB/BDNF pathway and Akt/Nrf2/Antioxidant enzyme in neuronal cells. Redox Biol, 11:592-599. 28110215 10.1016/j.redox.2016.12.034 PMC5247570

[54] Alghamdi BS (2018). The neuroprotective role of melatonin in neurological disorders. J Neurosci Res, 96:1136-1149. 29498103 10.1002/jnr.24220 PMC6001545

[55] Hardeland R (2018). Melatonin and inflammation-Story of a double-edged blade. J Pineal Res, 65:e12525. 30242884 10.1111/jpi.12525

[56] Miller E, Morel A, Saso L, Saluk J (2015). Melatonin redox activity. Its potential clinical applications in neurodegenerative disorders. Curr Top Med Chem, 15:163-169. 25985818

[57] Lacoste B, Angeloni D, Dominguez-Lopez S, Calderoni S, Mauro A, Fraschini F, et al. (2015). Anatomical and cellular localization of melatonin MT1 and MT2 receptors in the adult rat brain. J Pineal Res, 58:397-417. 25726952 10.1111/jpi.12224

[58] Fox JH, Kama JA, Lieberman G, Chopra R, Dorsey K, Chopra V, et al. (2007). Mechanisms of copper ion mediated Huntington's disease progression. PLoS One, 2:e334. 17396163 10.1371/journal.pone.0000334 PMC1828629

[59] Cavaleri F (2015). Review of Amyotrophic Lateral Sclerosis, Parkinson's and Alzheimer's diseases helps further define pathology of the novel paradigm for Alzheimer's with heavy metals as primary disease cause. Med Hypotheses, 85:779-790. 26604027 10.1016/j.mehy.2015.10.009

[60] Galano A, Tan DX, Reiter RJ (2013). On the free radical scavenging activities of melatonin's metabolites, AFMK and AMK. J Pineal Res, 54:245-257. 22998574 10.1111/jpi.12010

[61] Reiter RJ, Mayo JC, Tan DX, Sainz RM, Alatorre-Jimenez M, Qin L (2016). Melatonin as an antioxidant: under promises but over delivers. J Pineal Res, 61:253-278. 27500468 10.1111/jpi.12360

[62] Simunkova M, Alwasel SH, Alhazza IM, Jomova K, Kollar V, Rusko M, et al. (2019). Management of oxidative stress and other pathologies in Alzheimer's disease. Arch Toxicol, 93:2491-2513. 31440798 10.1007/s00204-019-02538-y

[63] Cao B, Chen Y, Ren Z, Pan Z, McIntyre RS, Wang D (2021). Dysregulation of kynurenine pathway and potential dynamic changes of kynurenine in schizophrenia: A systematic review and meta-analysis. Neurosci Biobehav Rev, 123:203-214. 33513412 10.1016/j.neubiorev.2021.01.018

[64] Almulla AF, Vasupanrajit A, Tunvirachaisakul C, Al-Hakeim HK, Solmi M, Verkerk R, et al. (2022). The tryptophan catabolite or kynurenine pathway in schizophrenia: meta-analysis reveals dissociations between central, serum, and plasma compartments. Mol Psychiatry, in press. 10.1038/s41380-022-01552-4 35422466

[65] Kindler J, Lim CK, Weickert CS, Boerrigter D, Galletly C, Liu D, et al. (2020). Dysregulation of kynurenine metabolism is related to proinflammatory cytokines, attention, and prefrontal cortex volume in schizophrenia. Mol Psychiatry, 25:2860-2872. 30940904 10.1038/s41380-019-0401-9 PMC7577855

[66] Erhardt S, Schwieler L, Imbeault S, Engberg G (2017). The kynurenine pathway in schizophrenia and bipolar disorder. Neuropharmacology, 112:297-306. 27245499 10.1016/j.neuropharm.2016.05.020

[67] Erhardt S, Blennow K, Nordin C, Skogh E, Lindström LH, Engberg G (2001). Kynurenic acid levels are elevated in the cerebrospinal fluid of patients with schizophrenia. Neurosci Lett, 313:96-98. 11684348 10.1016/s0304-3940(01)02242-x

[68] Buck SA, Baratta AM, Pocivavsek A (2020). Exposure to elevated embryonic kynurenine in rats: Sex-dependent learning and memory impairments in adult offspring. Neurobiol Learn Mem, 174:107282. 32738461 10.1016/j.nlm.2020.107282 PMC7506508

[69] Javitt DC, Zukin SR, Heresco-Levy U, Umbricht D (2012). Has an angel shown the way? Etiological and therapeutic implications of the PCP/NMDA model of schizophrenia. Schizophr Bull, 38:958-966. 22987851 10.1093/schbul/sbs069 PMC3446214

[70] Fazio F, Lionetto L, Curto M, Iacovelli L, Cavallari M, Zappulla C, et al. (2015). Xanthurenic Acid Activates mGlu2/3 Metabotropic Glutamate Receptors and is a Potential Trait Marker for Schizophrenia. Sci Rep, 5:17799. 26643205 10.1038/srep17799 PMC4672300

[71] Ulivieri M, Wierońska JM, Lionetto L, Martinello K, Cieslik P, Chocyk A, et al. (2020). The Trace Kynurenine, Cinnabarinic Acid, Displays Potent Antipsychotic-Like Activity in Mice and Its Levels Are Reduced in the Prefrontal Cortex of Individuals Affected by Schizophrenia. Schizophr Bull, 46:1471-1481. 32506121 10.1093/schbul/sbaa074 PMC7846105

[72] Oxenkrug G, van der Hart M, Roeser J, Summergrad P (2016). Anthranilic Acid: A Potential Biomarker and Treatment Target for Schizophrenia. Ann Psychiatry Ment Health, 4. PMC4817843 27042691

[73] Steardo L, Jr., Manchia M, Carpiniello B, Pisanu C, Steardo L, Squassina A (2020). Clinical, genetic, and brain imaging predictors of risk for bipolar disorder in high-risk individuals. Expert Rev Mol Diagn, 20:327-333. 32054361 10.1080/14737159.2020.1727743

[74] Benedetti F, Aggio V, Pratesi ML, Greco G, Furlan R (2020). Neuroinflammation in Bipolar Depression. Front Psychiatry, 11:71. 32174850 10.3389/fpsyt.2020.00071 PMC7054443

[75] Sellgren CM, Kegel ME, Bergen SE, Ekman CJ, Olsson S, Larsson M, et al. (2016). A genome-wide association study of kynurenic acid in cerebrospinal fluid: implications for psychosis and cognitive impairment in bipolar disorder. Mol Psychiatry, 21:1342-1350. 26666201 10.1038/mp.2015.186 PMC4965332

[76] Poletti S, Melloni E, Aggio V, Colombo C, Valtorta F, Benedetti F, et al. (2019). Grey and white matter structure associates with the activation of the tryptophan to kynurenine pathway in bipolar disorder. J Affect Disord, 259:404-412. 31610997 10.1016/j.jad.2019.08.034

[77] Beaumont V, Mrzljak L, Dijkman U, Freije R, Heins M, Rassoulpour A, et al. (2016). The novel KMO inhibitor CHDI-340246 leads to a restoration of electrophysiological alterations in mouse models of Huntington's disease. Exp Neurol, 282:99-118. 27163548 10.1016/j.expneurol.2016.05.005

[78] Dallaspezia S, Benedetti F (2009). Melatonin, circadian rhythms, and the clock genes in bipolar disorder. Curr Psychiatry Rep, 11:488-493. 19909672 10.1007/s11920-009-0074-1

[79] Kapczinski F, Frey BN, Zannatto V (2004). [Physiopathology of bipolar disorders: what have changed in the last 10 years?]. Braz J Psychiatry, 26 Suppl 3:17-21. 10.1590/s1516-44462004000700005 15597134

[80] Réus GZ, Jansen K, Titus S, Carvalho AF, Gabbay V, Quevedo J (2015). Kynurenine pathway dysfunction in the pathophysiology and treatment of depression: Evidences from animal and human studies. J Psychiatr Res, 68:316-328. 26028548 10.1016/j.jpsychires.2015.05.007 PMC4955923

[81] Oxenkrug G (2013). Serotonin-kynurenine hypothesis of depression: historical overview and recent developments. Curr Drug Targets, 14:514-521. 23514379 10.2174/1389450111314050002 PMC3726541

[82] Fuertig R, Azzinnari D, Bergamini G, Cathomas F, Sigrist H, Seifritz E, et al. (2016). Mouse chronic social stress increases blood and brain kynurenine pathway activity and fear behaviour: Both effects are reversed by inhibition of indoleamine 2,3-dioxygenase. Brain Behav Immun, 54:59-72. 26724575 10.1016/j.bbi.2015.12.020

[83] Al-Hakeim HK, Twayej AJ, Al-Dujaili AH, Maes M (2020). Plasma Indoleamine-2,3-Dioxygenase (IDO) is Increased in Drug-Naï ve Major Depressed Patients and Treatment with Sertraline and Ketoprofen Normalizes IDO in Association with Pro-Inflammatory and Immune- Regulatory Cytokines. CNS Neurol Disord Drug Targets, 19:44-54. 31894751 10.2174/1871527319666200102100307

[84] Smith AK, Simon JS, Gustafson EL, Noviello S, Cubells JF, Epstein MP, et al. (2012). Association of a polymorphism in the indoleamine- 2,3-dioxygenase gene and interferon-α-induced depression in patients with chronic hepatitis C. Mol Psychiatry, 17:781-789. 21691274 10.1038/mp.2011.67 PMC3179823

[85] Brundin L, Sellgren CM, Lim CK, Grit J, Pålsson E, Landén M, et al. (2016). An enzyme in the kynurenine pathway that governs vulnerability to suicidal behavior by regulating excitotoxicity and neuroinflammation. Transl Psychiatry, 6:e865. 27483383 10.1038/tp.2016.133 PMC5022080

[86] Strasser B, Gostner JM, Fuchs D (2016). Mood, food, and cognition: role of tryptophan and serotonin. Curr Opin Clin Nutr Metab Care, 19:55-61. 26560523 10.1097/MCO.0000000000000237

[87] Steinberg LJ, Underwood MD, Bakalian MJ, Kassir SA, Mann JJ, Arango V (2019). 5-HT1A receptor, 5-HT2A receptor and serotonin transporter binding in the human auditory cortex in depression. J Psychiatry Neurosci, 44:294-302. 31120232 10.1503/jpn.180190 PMC6710086

[88] Andrews PW, Bharwani A, Lee KR, Fox M, Thomson JA, Jr. (2015). Is serotonin an upper or a downer? The evolution of the serotonergic system and its role in depression and the antidepressant response. Neurosci Biobehav Rev, 51:164-188. 25625874 10.1016/j.neubiorev.2015.01.018

[89] Pocivavsek A, Wu HQ, Potter MC, Elmer GI, Pellicciari R, Schwarcz R (2011). Fluctuations in endogenous kynurenic acid control hippocampal glutamate and memory. Neuropsychopharmacology, 36:2357-2367. 21796108 10.1038/npp.2011.127 PMC3176574

[90] Haggarty SJ, Karmacharya R, Perlis RH (2021). Advances toward precision medicine for bipolar disorder: mechanisms & molecules. Mol Psychiatry, 26:168-185. 32636474 10.1038/s41380-020-0831-4 PMC10290523

[91] Fukuwatari T (2020). Possibility of Amino Acid Treatment to Prevent the Psychiatric Disorders via Modulation of the Production of Tryptophan Metabolite Kynurenic Acid. Nutrients, 12:1403. 32414200 10.3390/nu12051403 PMC7284450

[92] Pukos N, Goodus MT, Sahinkaya FR, McTigue DM (2019). Myelin status and oligodendrocyte lineage cells over time after spinal cord injury: What do we know and what still needs to be unwrapped? Glia, 67:2178-2202. 31444938 10.1002/glia.23702 PMC7217327

[93] Jacobs KR, Lovejoy DB (2018). Inhibiting the kynurenine pathway in spinal cord injury: Multiple therapeutic potentials? Neural Regen Res, 13:2073-2076. 30323124 10.4103/1673-5374.241446 PMC6199950

[94] Wang J, Jiang C, Zhang K, Lan X, Chen X, Zang W, et al. (2019). Melatonin receptor activation provides cerebral protection after traumatic brain injury by mitigating oxidative stress and inflammation via the Nrf2 signaling pathway. Free Radic Biol Med, 131:345-355. 30553970 10.1016/j.freeradbiomed.2018.12.014

[95] Qin D, Wang J, Le A, Wang TJ, Chen X, Wang J (2021). Traumatic Brain Injury: Ultrastructural Features in Neuronal Ferroptosis, Glial Cell Activation and Polarization, and Blood-Brain Barrier Breakdown. Cells, 10:1009. 33923370 10.3390/cells10051009 PMC8146242

[96] Meier TB, Nitta ME, Teague TK, Nelson LD, McCrea MA, Savitz J (2020). Prospective study of the effects of sport-related concussion on serum kynurenine pathway metabolites. Brain Behav Immun, 87:715-724. 32147388 10.1016/j.bbi.2020.03.002 PMC7316609

[97] Zhang R, Wang J, Huang L, Wang TJ, Huang Y, Li Z, et al. (2022). The pros and cons of motor, memory, and emotion-related behavioral tests in the mouse traumatic brain injury model. Neurol Res, 44:65-89. 34308784 10.1080/01616412.2021.1956290

[98] Li Q, Lan X, Han X, Durham F, Wan J, Weiland A, et al. (2021). Microglia-derived interleukin-10 accelerates post-intracerebral hemorrhage hematoma clearance by regulating CD36. Brain Behav Immun, 94:437-457. 33588074 10.1016/j.bbi.2021.02.001 PMC8058329

[99] Zhao W, Wu C, Stone C, Ding Y, Ji X (2020). Treatment of intracerebral hemorrhage: Current approaches and future directions. J Neurol Sci, 416:117020. 32711191 10.1016/j.jns.2020.117020

[100] Ren H, Han R, Chen X, Liu X, Wan J, Wang L, et al. (2020). Potential therapeutic targets for intracerebral hemorrhage-associated inflammation: An update. J Cereb Blood Flow Metab, 40:1752-1768. 32423330 10.1177/0271678X20923551 PMC7446569

[101] Cogo A, Mangin G, Maïer B, Callebert J, Mazighi M, Chabriat H, et al. (2021). Increased serum QUIN/KYNA is a reliable biomarker of post-stroke cognitive decline. Mol Neurodegener, 16:7. 33588894 10.1186/s13024-020-00421-4 PMC7885563

[102] Mo X, Pi L, Yang J, Xiang Z, Tang A (2014). Serum indoleamine 2,3-dioxygenase and kynurenine aminotransferase enzyme activity in patients with ischemic stroke. J Clin Neurosci, 21:482-486. 24412293 10.1016/j.jocn.2013.08.020

[103] Cuartero MI, de la Parra J, García-Culebras A, Ballesteros I, Lizasoain I, Moro M (2016). The Kynurenine Pathway in the Acute and Chronic Phases of Cerebral Ischemia. Curr Pharm Des, 22:1060-1073. 25248805 10.2174/1381612822666151214125950 PMC4972938

[104] Sadanandan N, Cozene B, Cho J, Park YJ, Saft M, Gonzales-Portillo B, et al. (2020). Melatonin-A Potent Therapeutic for Stroke and Stroke-Related Dementia. Antioxidants (Basel), 9:672. 32731545 10.3390/antiox9080672 PMC7463751

[105] Zhu H, Wang Z, Yu J, Yang X, He F, Liu Z, et al. (2019). Role and mechanisms of cytokines in the secondary brain injury after intracerebral hemorrhage. Prog Neurobiol, 178:101610. 30923023 10.1016/j.pneurobio.2019.03.003

[106] Thilo F, Suess O, Liu Y, Tepel M (2011). Decreased expression of transient receptor potential channels in cerebral vascular tissue from patients after hypertensive intracerebral hemorrhage. Clin Exp Hypertens, 33:533-537. 21957871 10.3109/10641963.2011.561903

[107] Rojewska E, Ciapała K, Piotrowska A, Makuch W, Mika J (2018). Pharmacological Inhibition of Indoleamine 2,3-Dioxygenase-2 and Kynurenine 3-Monooxygenase, Enzymes of the Kynurenine Pathway, Significantly Diminishes Neuropathic Pain in a Rat Model. Front Pharmacol, 9:724. 30050435 10.3389/fphar.2018.00724 PMC6050382

[108] MartInez-Coria H, Arrieta-Cruz I, Cruz ME, López-Valdés HE (2021). Physiopathology of ischemic stroke and its modulation using memantine: evidence from preclinical stroke. Neural Regen Res, 16:433-439. 32985462 10.4103/1673-5374.293129 PMC7996012

[109] Veres G, Fejes-Szabó A, Zádori D, Nagy-Grócz G, László AM, Bajtai A, et al. (2017). A comparative assessment of two kynurenic acid analogs in the formalin model of trigeminal activation: a behavioral, immunohistochemical and pharmacokinetic study. J Neural Transm (Vienna), 124:99-112. 10.1007/s00702-016-1615-5 27629500

[110] Perrin FE, Noristani HN (2019). Serotonergic mechanisms in spinal cord injury. Exp Neurol, 318:174-191. 31085200 10.1016/j.expneurol.2019.05.007

[111] Aeinehband S, Brenner P, Ståhl S, Bhat M, Fidock MD, Khademi M, et al. (2016). Cerebrospinal fluid kynurenines in multiple sclerosis; relation to disease course and neurocognitive symptoms. Brain Behav Immun, 51:47-55. 26189678 10.1016/j.bbi.2015.07.016

[112] Sundaram G, Lim CK, Brew BJ, Guillemin GJ (2020). Kynurenine pathway modulation reverses the experimental autoimmune encephalomyelitis mouse disease progression. J Neuroinflammation, 17:176. 32505212 10.1186/s12974-020-01844-y PMC7276083

[113] Correale J (2020). Immunosuppressive Amino-Acid Catabolizing Enzymes in Multiple Sclerosis. Front Immunol, 11:600428. 33552055 10.3389/fimmu.2020.600428 PMC7855700

[114] Gargaro M, Vacca C, Massari S, Scalisi G, Manni G, Mondanelli G, et al. (2019). Engagement of Nuclear Coactivator 7 by 3-Hydroxyanthranilic Acid Enhances Activation of Aryl Hydrocarbon Receptor in Immunoregulatory Dendritic Cells. Front Immunol, 10:1973. 31481962 10.3389/fimmu.2019.01973 PMC6710348

[115] San Hernandez AM, Singh C, Valero DJ, Nisar J, Trujillo Ramirez JI, Kothari KK, et al. (2020). Multiple Sclerosis and Serotonin: Potential Therapeutic Applications. Cureus, 12:e11293. 33274166 10.7759/cureus.11293 PMC7707915

[116] Mondanelli G, Coletti A, Greco FA, Pallotta MT, Orabona C, Iacono A, et al. (2020). Positive allosteric modulation of indoleamine 2,3-dioxygenase 1 restrains neuroinflammation. Proc Natl Acad Sci U S A, 117:3848-3857. 32024760 10.1073/pnas.1918215117 PMC7035626

[117] Lemos H, Mohamed E, Ou R, McCardle C, Zheng X, McGuire K, et al. (2020). Co-treatments to Boost IDO Activity and Inhibit Production of Downstream Catabolites Induce Durable Suppression of Experimental Autoimmune Encephalomyelitis. Front Immunol, 11:1256. 32625215 10.3389/fimmu.2020.01256 PMC7311583

[118] Fernández O (2011). Oral laquinimod treatment in multiple sclerosis. Neurologia, 26:111-117. 21163185 10.1016/j.nrl.2010.07.027

[119] Füvesi J, Rajda C, Bencsik K, Toldi J, Vécsei L (2012). The role of kynurenines in the pathomechanism of amyotrophic lateral sclerosis and multiple sclerosis: therapeutic implications. J Neural Transm (Vienna), 119:225-234. 22258797 10.1007/s00702-012-0765-3

[120] Boros F, Vécsei L (2020). Progress in the development of kynurenine and quinoline-3-carboxamide-derived drugs. Expert Opin Investig Drugs, 29:1223-1247. 10.1080/13543784.2020.1813716 32819186

[121] Potter LE, Doolen S, Mifflin K, Tenorio G, Baker G, Taylor BK, et al. (2018). Antinociceptive Effects of the Antidepressant Phenelzine are Mediated by Context-Dependent Inhibition of Neuronal Responses in the Dorsal Horn. Neuroscience, 383:205-215. 29752984 10.1016/j.neuroscience.2018.04.047 PMC6398164

[122] Cambron M, Mostert J, D'Hooghe M, Nagels G, Willekens B, Debruyne J, et al. (2019). Fluoxetine in progressive multiple sclerosis: The FLUOX-PMS trial. Mult Scler, 25:1728-1735. 31218911 10.1177/1352458519843051

[123] Royea J, Martinot P, Hamel E (2020). Memory and cerebrovascular deficits recovered following angiotensin IV intervention in a mouse model of Alzheimer's disease. Neurobiol Dis, 134:104644. 31669735 10.1016/j.nbd.2019.104644

[124] Zádori D, Veres G, Szalárdy L, Klivényi P, Vécsei L (2018). Alzheimer's Disease: Recent Concepts on the Relation of Mitochondrial Disturbances, Excitotoxicity, Neuroinflammation, and Kynurenines. J Alzheimers Dis, 62:523-547. 29480191 10.3233/JAD-170929

[125] Chatterjee P, Zetterberg H, Goozee K, Lim CK, Jacobs KR, Ashton NJ, et al. (2019). Plasma neurofilament light chain and amyloid-β are associated with the kynurenine pathway metabolites in preclinical Alzheimer's disease. J Neuroinflammation, 16:186. 31601232 10.1186/s12974-019-1567-4 PMC6788092

[126] Rajda C, Galla Z, Polyák H, Maróti Z, Babarczy K, Pukoli D, et al. (2020). Cerebrospinal Fluid Neurofilament Light Chain Is Associated with Kynurenine Pathway Metabolite Changes in Multiple Sclerosis. Int J Mol Sci, 21:2665. 32290514 10.3390/ijms21082665 PMC7216195

[127] Massudi H, Grant R, Braidy N, Guest J, Farnsworth B, Guillemin GJ (2012). Age-associated changes in oxidative stress and NAD+ metabolism in human tissue. PLoS One, 7:e42357. 22848760 10.1371/journal.pone.0042357 PMC3407129

[128] van der Velpen V, Rosenberg N, Maillard V, Teav T, Chatton JY, Gallart-Ayala H, et al. (2021). Sex-specific alterations in NAD+ metabolism in 3xTg Alzheimer's disease mouse brain assessed by quantitative targeted LC-MS. J Neurochem, 159:378-388. 33829502 10.1111/jnc.15362 PMC8596789

[129] Marlatt MW, Bauer J, Aronica E, van Haastert ES, Hoozemans JJ, Joels M, et al. (2014). Proliferation in the Alzheimer hippocampus is due to microglia, not astroglia, and occurs at sites of amyloid deposition. Neural Plast, 2014:693851. 25215243 10.1155/2014/693851 PMC4157009

[130] Bonda DJ, Mailankot M, Stone JG, Garrett MR, Staniszewska M, Castellani RJ, et al. (2010). Indoleamine 2,3-dioxygenase and 3-hydroxykynurenine modifications are found in the neuropathology of Alzheimer's disease. Redox Rep, 15:161-168. 20663292 10.1179/174329210X12650506623645 PMC2956440

[131] van der Velpen V, Teav T, Gallart-Ayala H, Mehl F, Konz I, Clark C, et al. (2019). Systemic and central nervous system metabolic alterations in Alzheimer's disease. Alzheimers Res Ther, 11:93. 31779690 10.1186/s13195-019-0551-7 PMC6883620

[132] Chatterjee P, Goozee K, Lim CK, James I, Shen K, Jacobs KR, et al. (2018). Alterations in serum kynurenine pathway metabolites in individuals with high neocortical amyloid-β load: A pilot study. Sci Rep, 8:8008. 29789640 10.1038/s41598-018-25968-7 PMC5964182

[133] Ramírez-Ortega D, Ramiro-Salazar A, González-Esquivel D, Ríos C, Pineda B, Pérez de la Cruz V (2017). 3-Hydroxykynurenine and 3-Hydroxyanthranilic Acid Enhance the Toxicity Induced by Copper in Rat Astrocyte Culture. Oxid Med Cell Longev, 2017:2371895. 28831293 10.1155/2017/2371895 PMC5555010

[134] Balmik AA, Chinnathambi S (2018). Multi-Faceted Role of Melatonin in Neuroprotection and Amelioration of Tau Aggregates in Alzheimer's Disease. J Alzheimers Dis, 62:1481-1493. 29562506 10.3233/JAD-170900

[135] Havelund JF, Heegaard NHH, Færgeman NJK, Gramsbergen JB (2017). Biomarker Research in Parkinson's Disease Using Metabolite Profiling. Metabolites, 7:42 28800113 10.3390/metabo7030042 PMC5618327

[136] Chang KH, Cheng ML, Tang HY, Huang CY, Wu YR, Chen CM (2018). Alternations of Metabolic Profile and Kynurenine Metabolism in the Plasma of Parkinson's Disease. Mol Neurobiol, 55:6319-6328. 29294246 10.1007/s12035-017-0845-3

[137] Tutakhail A, Boulet L, Khabil S, Nazari QA, Coudoré F (2020). Neuropathology of Kynurenine Pathway of Tryptophan Metabolism. Curr Pharmacol Rep, 6: 8-23.

[138] Heilman PL, Wang EW, Lewis MM, Krzyzanowski S, Capan CD, Burmeister AR, et al. (2020). Tryptophan Metabolites Are Associated With Symptoms and Nigral Pathology in Parkinson's Disease. Mov Disord, 35:2028-2037. 32710594 10.1002/mds.28202 PMC7754343

[139] Lim CK, Fernández-Gomez FJ, Braidy N, Estrada C, Costa C, Costa S, et al. (2017). Involvement of the kynurenine pathway in the pathogenesis of Parkinson's disease. Prog Neurobiol, 155:76-95. 27072742 10.1016/j.pneurobio.2015.12.009

[140] Medinas DB, Rozas P, Martínez Traub F, Woehlbier U, Brown RH, Bosco DA, et al. (2018). Endoplasmic reticulum stress leads to accumulation of wild-type SOD1 aggregates associated with sporadic amyotrophic lateral sclerosis. Proc Natl Acad Sci U S A, 115:8209-8214. 30038021 10.1073/pnas.1801109115 PMC6094144

[141] Maes M, Leonard BE, Myint AM, Kubera M, Verkerk R (2011). The new '5-HT' hypothesis of depression: cell-mediated immune activation induces indoleamine 2,3-dioxygenase, which leads to lower plasma tryptophan and an increased synthesis of detrimental tryptophan catabolites (TRYCATs), both of which contribute to the onset of depression. Prog Neuropsychopharmacol Biol Psychiatry, 35:702-721. 21185346 10.1016/j.pnpbp.2010.12.017

[142] Iłzecka J, Kocki T, Stelmasiak Z, Turski WA (2003). Endogenous protectant kynurenic acid in amyotrophic lateral sclerosis. Acta Neurol Scand, 107:412-418. 12757473 10.1034/j.1600-0404.2003.00076.x

[143] Tan VX, Guillemin GJ (2019). Kynurenine Pathway Metabolites as Biomarkers for Amyotrophic Lateral Sclerosis. Front Neurosci, 13:1013. 31616242 10.3389/fnins.2019.01013 PMC6764462

[144] Lee JM, Tan V, Lovejoy D, Braidy N, Rowe DB, Brew BJ, et al. (2017). Involvement of quinolinic acid in the neuropathogenesis of amyotrophic lateral sclerosis. Neuropharmacology, 112:346-364. 27265569 10.1016/j.neuropharm.2016.05.011

[145] Boros FA, Klivényi P, Toldi J, Vécsei L (2019). Indoleamine 2,3-dioxygenase as a novel therapeutic target for Huntington's disease. Expert Opin Ther Targets, 23:39-51. 30449219 10.1080/14728222.2019.1549231

[146] Morton AJ, Middleton B, Rudiger S, Bawden CS, Kuchel TR, Skene DJ (2020). Increased plasma melatonin in presymptomatic Huntington disease sheep (Ovis aries): Compensatory neuroprotection in a neurodegenerative disease? J Pineal Res, 68:e12624. 31742766 10.1111/jpi.12624

[147] Veres G, Molnár M, Zádori D, Szentirmai M, Szalárdy L, Török R, et al. (2015). Central nervous system-specific alterations in the tryptophan metabolism in the 3-nitropropionic acid model of Huntington's disease. Pharmacol Biochem Behav, 132:115-124. 25773340 10.1016/j.pbb.2015.03.002

[148] Lewitt PA, Li J, Lu M, Beach TG, Adler CH, Guo L (2013). 3-hydroxykynurenine and other Parkinson's disease biomarkers discovered by metabolomic analysis. Mov Disord, 28:1653-1660. 23873789 10.1002/mds.25555

[149] Stoy N, Mackay GM, Forrest CM, Christofides J, Egerton M, Stone TW, et al. (2005). Tryptophan metabolism and oxidative stress in patients with Huntington's disease. J Neurochem, 93:611-623. 15836620 10.1111/j.1471-4159.2005.03070.x

[150] Tan L, Yu JT, Tan L (2012). The kynurenine pathway in neurodegenerative diseases: mechanistic and therapeutic considerations. J Neurol Sci, 323:1-8. 22939820 10.1016/j.jns.2012.08.005

[151] Parasram K (2018). Phytochemical treatments target kynurenine pathway induced oxidative stress. Redox Rep, 23:25-28. 28651456 10.1080/13510002.2017.1343223 PMC6748679

[152] Lalut J, Karila D, Dallemagne P, Rochais C (2017). Modulating 5-HT(4) and 5-HT(6) receptors in Alzheimer's disease treatment. Future Med Chem, 9:781-795. 28504917 10.4155/fmc-2017-0031

[153] Briggs R, Kennelly SP, O'Neill D (2016). Drug treatments in Alzheimer's disease. Clin Med (Lond), 16:247-253. 27251914 10.7861/clinmedicine.16-3-247 PMC5922703

[154] Toublet FX, Lalut J, Hatat B, Lecoutey C, Davis A, Since M, et al. (2021). Pleiotropic prodrugs: Design of a dual butyrylcholinesterase inhibitor and 5-HT(6) receptor antagonist with therapeutic interest in Alzheimer's disease. Eur J Med Chem, 210:113059. 33310288 10.1016/j.ejmech.2020.113059

[155] Maitre M, Klein C, Patte-Mensah C, Mensah-Nyagan AG (2020). Tryptophan metabolites modify brain Aβ peptide degradation: A role in Alzheimer's disease? Prog Neurobiol, 190:101800. 32360535 10.1016/j.pneurobio.2020.101800

[156] Paul R, Borah A (2016). L-DOPA-induced hyperhomocysteinemia in Parkinson's disease: Elephant in the room. Biochim Biophys Acta, 1860:1989-1997. 27318154 10.1016/j.bbagen.2016.06.018

[157] Venkatesan D, Iyer M, Narayanasamy A, Siva K, Vellingiri B (2020). Kynurenine pathway in Parkinson's disease-An update. eNeurologicalSci, 21:100270. 33134567 10.1016/j.ensci.2020.100270 PMC7585940

[158] Verdin E (2015). NAD in aging, metabolism, and neurodegeneration. Science, 350:1208-1213. 26785480 10.1126/science.aac4854

[159] Wyant KJ, Ridder AJ, Dayalu P (2017). Huntington's Disease-Update on Treatments. Curr Neurol Neurosci Rep, 17:33. 28324302 10.1007/s11910-017-0739-9

[160] Thevandavakkam MA, Schwarcz R, Muchowski PJ, Giorgini F (2010). Targeting kynurenine 3-monooxygenase (KMO): implications for therapy in Huntington's disease. CNS Neurol Disord Drug Targets, 9:791-800. 20942784 10.2174/187152710793237430

[161] Darlington LG, Forrest CM, Mackay GM, Smith RA, Smith AJ, Stoy N, et al. (2010). On the Biological Importance of the 3-hydroxyanthranilic Acid: Anthranilic Acid Ratio. Int J Tryptophan Res, 3:51-59. 22084587 10.4137/ijtr.s4282 PMC3195249

[162] Wurfel BE, Drevets WC, Bliss SA, McMillin JR, Suzuki H, Ford BN, et al. (2017). Serum kynurenic acid is reduced in affective psychosis. Transl Psychiatry, 7:e1115. 28463241 10.1038/tp.2017.88 PMC5534956

[163] Zhou Y, Zheng W, Liu W, Wang C, Zhan Y, Li H, et al. (2019). Cross-sectional relationship between kynurenine pathway metabolites and cognitive function in major depressive disorder. Psychoneuroendocrinology, 101:72-79. 30419374 10.1016/j.psyneuen.2018.11.001

[164] Allison DJ, Ditor DS (2015). Targeting inflammation to influence mood following spinal cord injury: a randomized clinical trial. J Neuroinflammation, 12:204. 26545369 10.1186/s12974-015-0425-2 PMC4636770

[165] Amantea D, Micieli G, Tassorelli C, Cuartero MI, Ballesteros I, Certo M, et al. (2015). Rational modulation of the innate immune system for neuroprotection in ischemic stroke. Front Neurosci, 9:147. 25972779 10.3389/fnins.2015.00147 PMC4413676

[166] Bastos MAV Jr, Oliveira Bastos PRH, Portella RB, Soares LFG, Conde RB, Rodrigues PMF Jr, et al. (2019). Pineal gland and schizophrenia: A systematic review and meta-analysis. Psychoneuroendocrinology, 104:100-114. 30831343 10.1016/j.psyneuen.2019.02.024

[167] Takaesu Y (2018). Circadian rhythm in bipolar disorder: A review of the literature. Psychiatry Clin Neurosci, 72:673-682. 29869403 10.1111/pcn.12688

[168] Valdés-Tovar M, Estrada-Reyes R, Solís-Chagoyán H, Argueta J, Dorantes-Barrón AM, Quero-Chávez D, et al. (2018). Circadian modulation of neuroplasticity by melatonin: a target in the treatment of depression. Br J Pharmacol, 175:3200-3208. 29512136 10.1111/bph.14197 PMC6057892

[169] Zhang Y, Zhang WX, Zhang YJ, Liu YD, Liu ZJ, Wu QC, et al. (2018). Melatonin for the treatment of spinal cord injury. Neural Regen Res, 13:1685-1692. 30136678 10.4103/1673-5374.238603 PMC6128058

[170] Long R, Zhu Y, Zhou S (2019). Therapeutic role of melatonin in migraine prophylaxis: A systematic review. Medicine (Baltimore), 98:e14099. 30653130 10.1097/MD.0000000000014099 PMC6370052

[171] Anderson G, Jacob A, Bellivier F, Geoffroy PA (2016). Bipolar Disorder: The Role of the Kynurenine and Melatonergic Pathways. Curr Pharm Des, 22:987-1012. 26654772 10.2174/1381612822666151214105314

[172] Doolin K, Allers KA, Pleiner S, Liesener A, Farrell C, Tozzi L, et al. (2018). Altered tryptophan catabolite concentrations in major depressive disorder and associated changes in hippocampal subfield volumes. Psychoneuroendocrinology, 95:8-17. 29787958 10.1016/j.psyneuen.2018.05.019

[173] Lovelace MD, Varney B, Sundaram G, Lennon MJ, Lim CK, Jacobs K, et al. (2017). Recent evidence for an expanded role of the kynurenine pathway of tryptophan metabolism in neurological diseases. Neuropharmacology, 112:373-388. 26995730 10.1016/j.neuropharm.2016.03.024

[174] Shah UH, González-Maeso J (2019). Serotonin and Glutamate Interactions in Preclinical Schizophrenia Models. ACS Chem Neurosci, 10:3068-3077. 30807107 10.1021/acschemneuro.9b00044 PMC12502096

[175] Chollet F, Rigal J, Marque P, Barbieux-Guillot M, Raposo N, Fabry V, et al. (2018). Serotonin Selective Reuptake Inhibitors (SSRIs) and Stroke. Curr Neurol Neurosci Rep, 18:100. 30353288 10.1007/s11910-018-0904-9

[176] Liu G, Chen S, Zhong J, Teng K, Yin Y (2017). Crosstalk between Tryptophan Metabolism and Cardiovascular Disease, Mechanisms, and Therapeutic Implications. Oxid Med Cell Longev, 2017:1602074. 28377795 10.1155/2017/1602074 PMC5362714

[177] Birner A, Platzer M, Bengesser SA, Dalkner N, Fellendorf FT, Queissner R, et al. (2017). Increased breakdown of kynurenine towards its neurotoxic branch in bipolar disorder. PLoS One, 12:e0172699. 28241062 10.1371/journal.pone.0172699 PMC5328271

[178] Hajsl M, Hlavackova A, Broulikova K, Sramek M, Maly M, Dyr JE, et al. (2020). Tryptophan Metabolism, Inflammation, and Oxidative Stress in Patients with Neurovascular Disease. Metabolites, 10:208 32438592 10.3390/metabo10050208 PMC7281607

[179] Sakurai M, Yamamoto Y, Kanayama N, Hasegawa M, Mouri A, Takemura M, et al. (2020). Serum Metabolic Profiles of the Tryptophan-Kynurenine Pathway in the high risk subjects of major depressive disorder. Sci Rep, 10:1961. 32029791 10.1038/s41598-020-58806-w PMC7005270

[180] Pukoli D, Polyák H, Rajda C, Vécsei L (2021). Kynurenines and Neurofilament Light Chain in Multiple Sclerosis. Front Neurosci, 15:658202. 34113231 10.3389/fnins.2021.658202 PMC8185147

[181] Hatano T, Saiki S, Okuzumi A, Mohney RP, Hattori N (2016). Identification of novel biomarkers for Parkinson's disease by metabolomic technologies. J Neurol Neurosurg Psychiatry, 87:295-301. 25795009 10.1136/jnnp-2014-309676

[182] Forrest CM, Mackay GM, Stoy N, Spiden SL, Taylor R, Stone TW, et al. (2010). Blood levels of kynurenines, interleukin-23 and soluble human leucocyte antigen-G at different stages of Huntington's disease. J Neurochem, 112:112-122. 19845828 10.1111/j.1471-4159.2009.06442.x

[183] Yu D, Tao BB, Yang YY, Du LS, Yang SS, He XJ, et al. (2015). The IDO inhibitor coptisine ameliorates cognitive impairment in a mouse model of Alzheimer's disease. J Alzheimers Dis, 43:291-302. 25079795 10.3233/JAD-140414

[184] Török N, Tanaka M, Vécsei L (2020). Searching for Peripheral Biomarkers in Neurodegenerative Diseases: The Tryptophan-Kynurenine Metabolic Pathway. Int J Mol Sci, 21: 9338 33302404 10.3390/ijms21249338 PMC7762583

[185] Oxenkrug G, van der Hart M, Roeser J, Summergrad P (2017). Peripheral Tryptophan - Kynurenine Metabolism Associated with Metabolic Syndrome is Different in Parkinson's and Alzheimer's Diseases. Endocrinol Diabetes Metab J, in press. PMC5747375 29292800

[186] Bai JH, Zheng YL, Yu YP (2021). Urinary kynurenine as a biomarker for Parkinson's disease. Neurol Sci, 42:697-703. 32661882 10.1007/s10072-020-04589-x

[187] Deng Y, Zhou M, Wang J, Yao J, Yu J, Liu W, et al. (2021). Involvement of the microbiota-gut-brain axis in chronic restraint stress: disturbances of the kynurenine metabolic pathway in both the gut and brain. Gut Microbes, 13:1-16. 10.1080/19490976.2020.1869501 PMC7872056 33535879

[188] Huang GL, Tao A, Miyazaki T, Khan T, Hong T, Nakagawa Y, et al. (2019). PEG-Poly(1-Methyl-l-Tryptophan)-Based Polymeric Micelles as Enzymatically Activated Inhibitors of Indoleamine 2,3-Dioxygenase. Nanomaterials (Basel), 9:719 31075929 10.3390/nano9050719 PMC6566635

[189] Schmidt SK, Siepmann S, Kuhlmann K, Meyer HE, Metzger S, Pudelko S, et al. (2012). Influence of tryptophan contained in 1-Methyl-Tryptophan on antimicrobial and immunoregulatory functions of indoleamine 2,3-dioxygenase. PLoS One, 7:e44797. 23028625 10.1371/journal.pone.0044797 PMC3441469

[190] Yu CP, Pan ZZ, Luo DY (2016). TDO as a therapeutic target in brain diseases. Metab Brain Dis, 31:737-747. 27072164 10.1007/s11011-016-9824-z

[191] Nematollahi A, Sun G, Jayawickrama GS, Hanrahan JR, Church WB (2016). Study of the Activity and Possible Mechanism of Action of a Reversible Inhibitor of Recombinant Human KAT-2: A Promising Lead in Neurodegenerative and Cognitive Disorders. Molecules, 21:856 27367665 10.3390/molecules21070856 PMC6273595

[192] Lu H, Kopcho L, Ghosh K, Witmer M, Parker M, Gupta S, et al. (2016). Development of a RapidFire mass spectrometry assay and a fluorescence assay for the discovery of kynurenine aminotransferase II inhibitors to treat central nervous system disorders. Anal Biochem, 501:56-65. 26874021 10.1016/j.ab.2016.02.003

[193] Noorbakhsh A, Hosseininezhadian Koushki E, Farshadfar C, Ardalan N (2021). Designing a natural inhibitor against human kynurenine aminotransferase type II and a comparison with PF-04859989: a computational effort against schizophrenia. J Biomol Struct Dyn, 40:7038-7051 33645449 10.1080/07391102.2021.1893817

[194] Jayawickrama GS, Nematollahi A, Sun G, Church WB (2018). Improvement of kynurenine aminotransferase-II inhibitors guided by mimicking sulfate esters. PLoS One, 13:e0196404. 29689093 10.1371/journal.pone.0196404 PMC5915280

[195] Zhang S, Collier MEW, Heyes DJ, Giorgini F, Scrutton NS (2021). Advantages of brain penetrating inhibitors of kynurenine-3-monooxygenase for treatment of neurodegenerative diseases. Arch Biochem Biophys, 697:108702. 33275878 10.1016/j.abb.2020.108702 PMC8111166

[196] Gao J, Yao L, Xia T, Liao X, Zhu D, Xiang Y (2018). Biochemistry and structural studies of kynurenine 3-monooxygenase reveal allosteric inhibition by Ro 61-8048. FASEB J, 32:2036-2045. 29208702 10.1096/fj.201700397RR

[197] Hughes TD, Güner OF, Iradukunda EC, Phillips RS, Bowen JP (2022). The Kynurenine Pathway and Kynurenine 3-Monooxygenase Inhibitors. Molecules, 27:273 35011505 10.3390/molecules27010273 PMC8747024

[198] Toledo-Sherman LM, Prime ME, Mrzljak L, Beconi MG, Beresford A, Brookfield FA, et al. (2015). Development of a series of aryl pyrimidine kynurenine monooxygenase inhibitors as potential therapeutic agents for the treatment of Huntington's disease. J Med Chem, 58:1159-1183. 25590515 10.1021/jm501350y

